# Quality improvement strategies at primary care level to reduce inequalities in diabetes care: an equity-oriented systematic review

**DOI:** 10.1186/s12902-018-0260-4

**Published:** 2018-05-29

**Authors:** Natalie Terens, Simona Vecchi, Anna Maria Bargagli, Nera Agabiti, Zuzana Mitrova, Laura Amato, Marina Davoli

**Affiliations:** 1 Trenton Health Team, Trenton, New Jersey USA; 2 Department of Epidemiology, Lazio Region- ASL Rome1, Rome, Italy

**Keywords:** Type 2 diabetes, Quality improvement strategies, Equity, Systematic review

## Abstract

**Background:**

There is evidence that disparities exist in diabetes prevalence, access to diabetes care, diabetes-related complications, and the quality of diabetes care. A wide range of interventions has been implemented and evaluated to improve diabetes care. We aimed to review trials of quality improvement (QI) interventions aimed to reduce health inequities among people with diabetes in primary care and to explore the extent to which experimental studies addressed and reported equity issues.

**Methods:**

Pubmed, EMBASE, CINAHL, and the Cochrane Library were searched to identify randomized controlled studies published between January 2005 and May 2016. We adopted the PROGRESS Plus framework, as a tool to explore differential effects of QI interventions across sociodemographic and economic factors.

**Results:**

From 1903 references fifty-eight randomized trials met the inclusion criteria (with 17.786 participants), mostly carried out in USA. The methodological quality was good for all studies. Almost all studies reported the age, gender/sex and race distribution of study participants. The majority of trials additionally used at least one further PROGRESS-Plus factor at baseline, with education being the most commonly used, followed by income (55%). Large variation was observed between these studies for type of interventions, target populations, and outcomes evaluated. Few studies examined differential intervention effects by PROGRESS-plus factors. Existing evidence suggests that some QI intervention delivered in primary care can improve diabetes-related health outcomes in social disadvantaged population subgroups such as ethnic minorities. However, we found very few studies comparing health outcomes between population subgroups and reporting differential effect estimates of QI interventions.

**Conclusions:**

This review provides evidence that QI interventions for people with diabetes is feasible to implement and highly acceptable. However, more research is needed to understand their effective components as well as the adoption of an equity-oriented approach in conducting primary studies. Moreover, a wider variety of socio-economic characteristics such as social capital, place of residence, occupation, education, and religion should be addressed.

**Electronic supplementary material:**

The online version of this article (10.1186/s12902-018-0260-4) contains supplementary material, which is available to authorized users.

## Background

Diabetes is a complex, chronic disease recognized as an important cause of premature death and disability [1] and disproportionately affects socially and economically disadvantaged populations [2–4]. According the National Institute for Health and Care Excellence guidelines [5], patients with type 2 diabetes should receive a clear gamut of care to be provided by primary care providers. Annual routine monitoring of health indicators such as urinary albumin, BMI, cholesterol, blood creatinine, HbA1c and BP measured, eyes and feet examined and a smoking review, forms a major part of patient diabetes care. In addition patients should expect to receive an evidenced-based education and access to specialist healthcare professionals including ophthalmologists, podiatrists and dieticians.

Quality of care among diabetic patient can be influenced by a range of factors that has been already described. Previous systematic reviews showed that low individual socio-economic status and residential area deprivation are often associated with both worse process indicators and worse intermediate outcomes among patients with type 2 diabetes [6]. These differences are present even in countries with a significant level of economic development that have a universal health care system. Moreover, disparities in diabetes care exist among racial or ethnic minority groups, independent of economic status [7].

To improve diabetes care, it might be important to focus on quality management (QM), especially because the complexity of healthcare system and patients complexities has dramatically increased. QM comprises procedures to monitor, assess, and enhance the quality of care. In the last years many countries have developed quality improvement interventions (QI) to improve both patient outcomes and the quality of diabetes care [8, 9]. A meta-analysis of studies investigating QI strategies [10] found that interventions targeting the entire system of disease management (team changes, case management, promotion of self-management) along with patient-mediated QI activities were important components of strategies to improve diabetes care. However, the studies included in this review were targeted to the general population, irrespective of socio-demographic characteristics or socio-economic status.

Acknowledging the existence of such disparities, our aims are to: a) describe the extent to which effects on social inequalities are considered in randomized controlled trials (RCTs) evaluating the effects of QI interventions to improve quality of diabetes care and b) synthesize evidence on the effectiveness of QI strategies to reduce health inequities in diabetes care in the primary care setting. We conducted an equity-oriented systematic review including RCTs only, using an international taxonomy of QI interventions, and assessing the quality of included studies with a methodological rating tool.

## Methods

For the purpose of the review, a “socially disadvantaged group” is defined by differences that place the group at distinct levels in a social hierarchy. To explicitly consider health equity and to capture characteristics possibly indicating disadvantaged status, we adopted the PROGRESS-Plus framework recommended by the Campbell and Cochrane Equity Methods Group and the Cochrane Public Health Group to identify studies with a focus on reducing health inequalities [11]*.* PROGRESS-Plus stands for place of residence, race/ethnicity/culture/language, occupation, gender/sex, religion, socioeconomic status and social capital. This systematic review was conducted in accordance with PRISMA-E 2012 (Preferred Reporting Items for Systematic Reviews and Meta-Analyses, Equity 2012 Extension), a validated tool to improve both the reporting and conducting of equity focused systematic reviews, were upheld in this review [12].

### Data sources and searches

We searched all relevant biomedical databases such as Pubmed, EMBASE, CINAHL, and the Cochrane Library for relevant published RCTs and cluster-RCTs published in English. We limited the search from 1 January 2005 to 31 May 2016. A combination of MeSH terms and keywords were chosen to reflect selection criteria tailored to each database. Details of the full search strategy for PubMed are included in supplemental material (Additional file 1). In addition, we scanned the reference lists of relevant reviews to track relevant RCTs.

### Study selection

Two authors (NT, AMB) independently screened all title and abstracts of all studies obtained from electronic searches. For studies meeting the inclusion criteria, we retrieved full texts and the same authors independently evaluated them for inclusion. Any disagreements were resolved through consensus or in discussion with the extended authorial group.

We used the “population, intervention, comparison, outcome, setting” (PICOS) logic to guide the systematic review (Additional file 2). We included randomized controlled trials (RCTs) and cluster-randomized trials, evaluating all QI interventions designed to improve health outcomes in social disadvantaged people with type 2 diabetes and designed to reduce inequalities in diabetes care. We considered studies that reported quantitative estimates of total effect of treatment and differential effects for the PROGRESS-Plus factors.

We used the Agency for Healthcare Research and Quality [13] taxonomy to identify QI strategies (Additional file 3). QI strategies can be delivered to specific levels of influence:

- Patient level (e.g. patient education, patient reminders, or promotion of self-management);
- Health care provider level (e.g. electronic medical record reminders, audit & feedback, cultural competency training);
- Health care system level (e.g. change in the health system structure or delivery, adjusting roles of care team members, nurse care management model).

### Data extraction and quality assessment

Two authors independently extracted data (NT, SV), and disagreements were resolved by discussion. Data from multiple publications of the same study was considered as a single study. A data extraction form was designed to document the following study details: trials characteristics; participants (total number at baseline, age range, gender, clinical features); type of intervention and comparator; clinical and no clinical outcomes; timing; risk of bias; study results. For continuous outcomes, we extracted the mean change from baseline (with the standard deviation) and the mean difference, if available, with the corresponding 95% confidence interval (CIs). Relative risk (RR), and absolute risk differences, with the corresponding 95% CI, was extracted for binary primary outcomes. If studies reported data for more than one time point, we extracted data for the longest-term outcomes.

Baseline population characteristics relevant for addressing potential issues in health equity were extracted using the PROGRESS-Plus framework. We extracted data on outcome assessed, according to whether PROGRESS-Plus factors were considered as control variables (e.g., by adjusting in regression analyses) and the methods utilized to investigate differential effects (stratified analysis or modification/interaction analysis). We also extracted details on the duration of intervention, duration of follow up, health professional group involved, details of the strategy being implemented (i.e. modality, delivery format).

Two authors independently assessed risk of bias of included studies using the Cochrane ‘Risk of bias’ tool for RCTs [14]. We considering the following domains: sequence generation, allocation concealment, blinding of participants and personnel, blinding of outcome assessment, incomplete data, selective reporting, and other biases. For each domain, risk of bias was classified as “high,” “low,” or “unclear”. Since we included cluster-randomized controlled trials, additional items were considered: (1) recruitment bias: did recruitment of diabetes patients take place before or after randomization of the clusters?, (2) did the intervention and control group differ in baseline characteristics?, (3) did any of the clusters drop out during follow-up, (4) was clustering accounted for in the statistical analyses? We investigated detection bias separately for objective and subjective outcome measures. We defined clinical and laboratory measures, process indicators, diabetes complications, hospital admissions, emergency admissions and all-cause mortality as objective outcome measures. We defined measures of self-management/adherence to recommendations as subjective outcome measures. With respect to missing data, we judged individual trials at high risk of bias if data from more than 10% of participants were not available. We used the quality criteria for descriptive purposes only to highlight differences between studies. We used RevMan 2014 software [15] to generate figures related to risk of bias.

### Data synthesis

We synthesized findings from the included studies by intervention level (patients, health care provider, and health care system). The wide variety of interventions (in terms of mode of delivery, frequency and duration of follow up assessment) and population groups considered in the included studies did not allow for a meaningful meta-analysis to be conducted. We summarized results using narrative methods. We described in more detail studies reporting differences in QI interventions effects across subgroups.

## Results

**Fig. 1:**
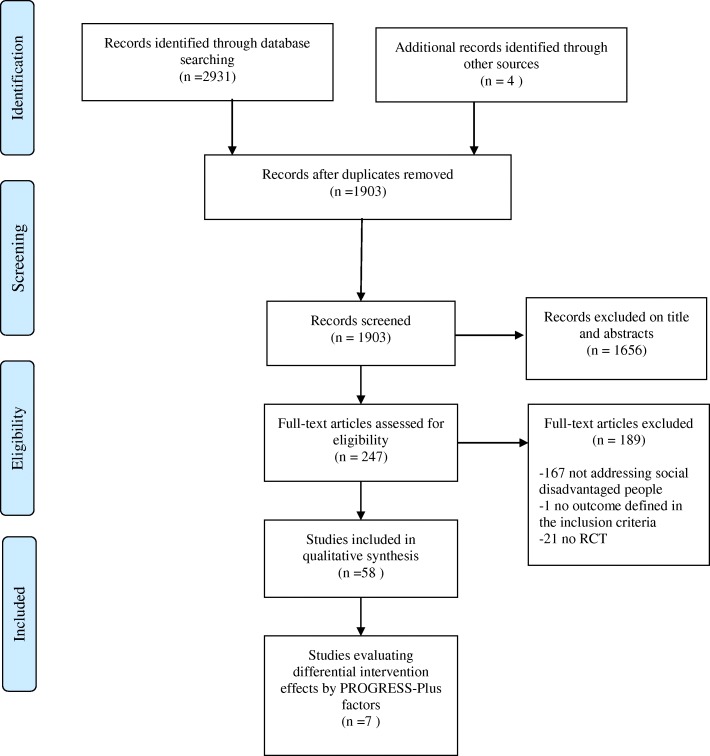
PRISMA 2009 Flow Diagram. Study selection process

The search strategy generated 1903 citations after removing duplicates. Upon reviewing titles and abstracts, we retrieved full text articles for 247 studies that were screened by two authors independently (NT, AMB). We excluded 189 trials. Most common reasons for exclusion were not addressing a socially disadvantaged group, an evaluation of primary prevention intervention, and being conducted in a setting other than primary care. Fifty-eight RCTs met eligibility criteria. PRISMA Flow Diagram Fig. 1 shows the details of study selection process.

### Overview of the included studies

A substantial synthesis of the characteristics of all 58 studies included in this review is reported in Table 1. Overall the majority of studies (*n* = 54) used a parallel RCT design while four trials were cluster RCTs [16–19]. Follow-up periods varied in duration from less than 1 month to 5 years, with the majority lasting 6 to 12 months. Most of trials were conducted in the USA (*n* = 47); the remaining studies were carried out in Canada [20], Asia [21], the United Kingdom [16], New Zealand [22], Australia [19], Trinidad and American Samoa [18, 23].

**Table 1:** Synthesis of the characteristics of the included studies by level of intervention and PROGRESS factors

Level of intervention	Patient level		Provider level		Health care systems level		Total QI strategies	
Total of studies	29		3		26		58	
	N	%	N	%	N	%	N	%
Sample characteristics								
Age	55.13	-	55.37	-	53.82	-	55.06	-
Sex, female (%)		64.05		58.69		57.84		60.20
Baseline HgA1c (%; mmol/mol)	8.88; 74	7.0–11.8; 53–105	9.53; 81	8.1–12.05; 31–109	8.51; 70	7.6–10.5; 60–91	8.88; 74	7.0–12.05; 53–109
Progress factors reported at baseline								
Place of residence	29	50	3	5.2	26	44.8	58	-
Race/ethnicity	26	49.1	3	5.7	24	45.3	53	-
Occupation	12	54.5	–	-	10	45.5	22	-
Gender/sex	24	46.2	3	5.8	25	48.1	52	-
Religion	–	-	–	-	–	-	–	-
Education	26	57.7	1	2.2	18	40.1	45	-
Socioeconomic status (SES)	–	-	–	-	–	-	–	-
Income	20	62.5	–	-	12	37.5	32	-
Social capital	10	62.5	-	-	6	37.5	16	-
Age	28	50.0	3	5.4	25	44.7	56	-
Disability	–	-	–	-	–	-	–	-
Sexual orientation	–	-	–	-	–	-	–	-
Study characteristics								
Year of publication								
2005–2010	11	19	2	3.5	11	19	24	41.4
2011–2016	18	31	1	1.7	15	25.9	34	58.6
Study location								
North America	25	86.2	3	100	22	85	47	
UK	1	3.4	–	-	1	3.8	2	3.4
Australia	–	–	–	-	2	-	2	3.4
Asia	3	10.4	–	-	1	7.7	4	6.9
Duration of study (months)	10	3–26	4.5	0.25–36	12	6–60	8.9	0.25–60
Average sample size (range)	190 (56–526)		1573 (182–4138)		290 (65–1665)		684 (50–4138)	

Almost all studies reported the age, gender/sex and race distribution of study participants. The majority of studies additionally used at least one further PROGRESS-Plus factor for the description of participants’ baseline characteristics.

Among these, education was the most commonly reported factor (*n* = 45), followed by income (*n* = 32). Twenty-six studies considered at least one PROGRESS-Plus factor as control variable when measuring intervention effects (e.g., by adjusting in multivariate analyses). Again, age (*n* = 23) and gender/sex (*n* = 20) were the factors most commonly controlled for, followed by education (*n* = 9). Seven (12%) trials used at least one PROGRESS-Plus factors for examining differential intervention effects, and gender, age, race and education were those most often considered.

Detailed descriptions of the QI interventions were not always clearly provided in the trials. In order of frequency, were twenty-nine studies (50%) focused on interventions delivered at the patient level [17, 20, 21, 24–27, 29, 32–39, 41, 42, 55, 61–70], and twenty-six at the health care organization level (45%) [16, 18, 19, 22, 23, 28, 30, 31, 40, 45–54, 56–60, 72, 73]. The remaining three studies (5%) [43, 44, 71] described interventions at the provider level. In the majority of studies comparators were “usual” or “standard” care (69%), five studies reported waiting list, delayed intervention or no intervention. Health professionals who participated in studies included physicians, specialist nurses, social workers, dietitians, diabetes educators, community health workers, general practitioners, practice nurses and home care nurses.

The majority of trials (96%) provided data on change in HbA1c. Thirty-seven trials (63%) reported BMI outcome; blood pressure and cholesterol data in 38 and 30 trials, respectively. Process measures including diabetic foot exam, dilated eye exam and attendance at office appointments were seldom reported.

For secondary outcomes, data were available for patient-reported measures including diet and physical activity (*n* = 28) using a considerable variety of instruments. Medication adherence and home glucose monitoring were measured less consistently (in 17 and 15 studies, respectively) as were diabetes complications and hospital admissions.

A detailed description of trials characteristics and intervention components by intervention level is presented in Additional file 4.

### Risk of bias in included studies

**Fig. 2:**
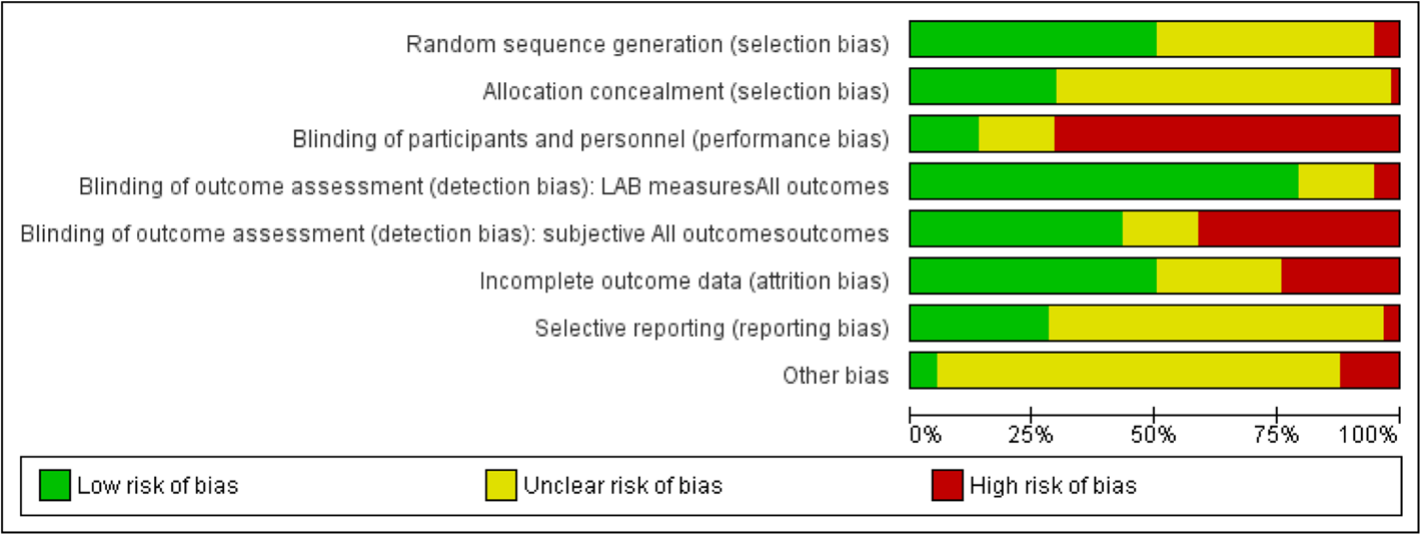
Risk of bias graph

**Fig. 3:**
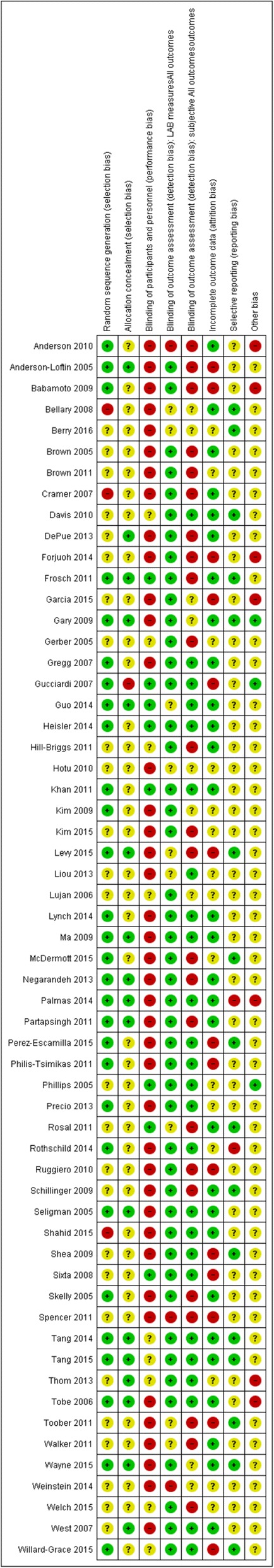
Risk of bias summary

A summary of ‘risk of bias’ for each study and comparative data across the studies is reported in Figs. 2 and 3 . All studies were described as individual RCT (*n* = 54) or cluster-RCTs (*n* = 4). None of the randomized studies had uniformly low risk of bias. The allocation sequence was adequately reported in 48% of the studies (28/58), with random number tables or a computer-generated randomized list as the most commonly used methods. One study was categorized as high risk due to the use of a gender-based randomization procedure [24]. Most RCTs (40/58) did not describe or described in sufficient detail the allocation concealment to allow a judgment and were evaluated to be at unclear risk of bias.

In the majority of the trials, all participants were aware of the treatment they were receiving, and only eight studies blinded providers [20, 21, 25–30]. For studies reporting objective outcomes with standardized collection methods (e.g. automated blood test), we assigned a low risk of detection bias (79%), as knowledge of treatment assignment was considered unlikely to affect the outcome. Twenty-eight studies reporting subjective outcomes, those that used self-reported measures (i.e. questionnaire on dietary habits or physical activities) were at high risk of bias due to the lack of blinding of outcome assessment (24 studies). In the remaining 30 studies, independent research personnel who were not involved in the intervention performed outcome assessments, which we evaluated as low risk of detection bias.

Thirty studies were at low risk of incomplete outcome data due to a low attrition rate (< 10%) or an intention-to-treat (ITT) analysis for primary outcomes. Thirteen studies were at high risk of bias because a high proportion of participants were lost to follow-up or were missing outcome measurements. Selective reporting bias was difficult to detect in most studies because published protocols were often unavailable. Most trials reported all outcomes. One study [30] collected a large quantity of baseline data but did not adequately describe follow-up data. One paper [31] did not report some subjective measures listed in the published protocol. Risk of contamination was high in most of the studies because patients receiving interventions and those receiving usual care or other interventions were seen within the same health center. Among cluster RCTs, three accounted for the effects of clustering in their results analysis.

### Study evaluating the effect of QI strategies by intervention level (*n* = 51)

#### Patient level

More than half (*n* = 17) of the studies showed significant effect in at least one of the outcomes considered in this review; most (*n* = 11) of these interventions include group education sessions or visits and principles of self-management.

Twenty-seven out of 29 trials reported data on glycemic control measured as HbA1c level. Ten studies reported an improvement in HbA1c levels in the experimental group compared to the control group.

An education program based on telephone calls [32] was found to be associated with a decrease in HbA1c both in the unadjusted (− 0.23 ± 0.11% vs 0.13 ± 0.13%, *p* < 0.04, *n* = 526) and adjusted analysis (MD = 0.40, 95% CI 0.10–0.70; *p* = 0.009).

Rosal et al. [27] evaluated a nutritionist or health educator-led self-management education program supported by counseling and a self-monitoring device. The study showed a difference between groups in HbA1c level at 4 months (MD = − 0.53, 95% CI-0.92 to − 0.14; *p* > 0.008, *n* = 252) but not sustained at 12 months.

An intensive training group intervention addressing both diabetes and cardiovascular diseases, combined with problem-solving training sessions [29], was effective in improving glycemic control (MD = − 0.72, 95% CI − 1.42 to − 0.01, *p* = 0.02, *n* = 56).

Two studies (*n* = 265) showed an improvements in glycemic control as measured by HbA1c (8.2% ± 0.4 vs 8.6% ±0.3, *p* = 0.004 and 7.6 ± 1.8 vs 8.2 ± 2.5; *p* = 0.006, respectively), comparing behavioral education programs via telehealth [33] or using a computerized self-management program [26] vs standard care.

Berry et al. [17] reported a greater improvement in HbA1c levels in low-income participants receiving sessions led by a multidisciplinary team than in the control group (7.6% vs 9.3%; *p* = 0.001, *n* = 80).

One study [21] found that an education program with incentives and self-monitoring devices produced a significant reduction in HbA1c (7.29% ±0.58 vs 7.73% ±0.57; *p* < 0.05, *n* = 132).

Philis-Tsimikas et al. [34] did not report difference between groups but a significant decrease of HbA1c from baseline to follow-up (− 1.5%, *p* < 0.01) was observed in the experimental group.

Finally, two trials [35, 36] did not find a significant decrease in HbA1c in the study population, but reported a positive association for a subgroup of participants. Brown et al. [35] (*n* = 460) found that for those who attended ≥50% of the self-management patient education sessions, the reduction of HbA1c was − 0.6% for the “compressed” group and − 1.7% for the “extended” group. In Gerber et al. [36] (*n* = 244), the intervention resulted in significant improvement in HbA1c among low–health literacy subjects with poor glycemic control.

Eighteen trials reported data on change in BMI, three found a significant improvement in the experimental group.

Anderson-Loftin et al. [37] reported that the group exposed to the dietary self-management intervention had a decrease in BMI while the control group showed an increase in BMI control group (− 0.81 kg/m2 vs + 0,57 Kg/m^2^; *p* = 0.009, *n* = 97). Tang et al. [38] reported a decrease in BMI in the intervention group receiving behavioral support delivered by a peer leader compared with the control group; the benefit was observed at different follow-up times and maintained at the longest one (15 months) (MD = − 0.8 Kg/m^2^ 95CI%-1.6 to − 0.1; *p* = 0.032, *n* = 106). Toobert et al. [39] showed a significant difference in BMI (MD of − 0.40 Kg/m^2^; *p* < 0.05, *n* = 280) in an underserved and high-risk Latino population treated with a long-term multiple-behavior-change program.

Fifteen of the 26 studies examining healthcare interventions in diabetes care considered blood pressure among the outcomes. Two studies showed differences favoring the experimental intervention. In the study conducted by Hill-Briggs et al. [29], participants receiving a self-management training adapted for low literacy experienced an individual improvement in DBP and SBP (median reduction = − 7.17 mmHg, *n* = 8, median reduction of − 14.67 mmHg, *n* = 9, respectively). Tang et al. [40] also reported a greater reduction in the group that received a combination of self-management and peer support interventions than the control group, both in SBP (MD = − 10.0 mmHg (95% CI -17.6 to − 2.4, *p* = 0.01) and DBP (MD = − 8.3 mmHg (95% CI -13.2 to − 3.4, *p* = 0 .001).

A significant improvement (*p* < 0.001) in hypertension in both groups was found by Shahid et al. [24] (*n* = 440) but between-group differences were not reported.

Eighteen studies reported data on diet adherence. Seven studies [22, 25, 31, 35, 39, 44, 51] observed between group differences although using different instruments and scales.

Anderson-Loftin et al. [37] used the Food Habits Questionnaire (FHQ) adapted for southern African Americans to measure dietary pattern. The intervention was a patient education program delivered by nurse case manager with nutrition focus combined with support groups, and weekly telephone follow-up. The authors reported a significant improvement in the experimental group with a decrease in high-fat diet while the control group continued previous high-fat dietary behaviors (MD =0.2 points, *p* = 0.005).

One trial [20] used the Summary of Diabetes Self-care Activities Questionnaire (SDCA) to assess the nutrition adherence in Canadian Portuguese-speaking adults. There was an improvement in self-reported nutrition adherence at 3 months in favor of the experimental intervention (MD = 0.42 ± 0.14, *p* < 0.05, *n* = 87).

Negarandeh [41] evaluated patient education program based on different format (Pictorial or teach back strategy) compared to usual care. Adherence to dietary pattern was measured through a self-structured nine-item scale. The score improved in all study participants (*n* = 130) in follow up measurements but the improvement was more pronounced for the intervention groups than the control group (*p* < 0.05). The mean difference between groups was − 2.24 (95% CI- 2.67 to-1.81) for the Pictorial format group, and − 2.52 (95% CI:-2.95 to − 2.09) for the Teach back format group.

A culturally tailored self-management intervention adapted for a low income Latino group [27], improved the quality of diet as measured by the Alternative Healthy Eating Index. Significant between group differences were found at 12 months (MD = 2.83 95% CI 0.58 to 5.08, *p* = 0.014, *n* = 252).

A similar intervention was evaluated by Shahid et al. [24] among people residing in rural areas in Pakistan. In the intervention group there was a significant increase in the proportion of participants compliant to the diet plan (17.3% at baseline to 43.6% at follow up, *p* < 0.01) while in the control group there was no significant increase (13.6% at baseline to 15.9% follow up, *p* = 0.522).

Weinstein’s trial [42] assessed fruit and vegetable consumption self-reported daily following brief educational intervention. At 12 weeks, the percentage of participants who reported ever purchasing from a produce market increased significantly in the intervention group (81% vs 48%; *p* = 0.003, *n* = 79). Moreover, there was an overall decrease of the percentage of participants reporting difficulty affording fresh fruits and vegetables (55% vs 74% at baseline, *p* = 0.008). This decrease was not significantly different between arms.

Toobert et al. [39] reported the percent of calories from saturated fat measured using a food frequency questionnaire following a culturally adapted Mediterranean lifestyle intervention. He found an improvement of 0.33 points at the 24-month follow-up.

#### Provider level

Two studies evaluating reminder and reminder+feedback interventions [43, 44] showed an improvement in glycemic control (HbA1c) compared to the usual care or no intervention group (0.6% vs 0.2%, *p* < 0.02, *n* = 399; MD = − 0.80 *p* < 0.001, *n* = 2046, respectively). Both of these interventions utilized computerized systems to produce physician reminders. One study [43] found an improvement for LDL cholesterol for all intervention arms, with the greater change observed in the reminders+feedback group (− 18 mg/dl). No studies reported differences between intervention and control arms for blood pressure and BMI.

#### Health care system level

The majority of studies that evaluated interventions targeting the health care system (*n* = 20), showed significant effect in at least one of the outcomes considered in this review.

As far HbA1c, nine studies reported a significant reduction of HbA1c values [18, 23, 30, 45–50] with a mean difference ranging from − 0.29% to − 0.8%. The studies considered a range of health care system-based strategies including interventions such as individualized case management activities [23], and culturally tailored counseling delivered by a CHW [46, 47, 49]^2^ and/or NCM [18, 45], and promotoras [50]. Three RCTs included additional activities, in particular home visits to support patient’s progress [30, 47, 48].

Seven studies found a significantly greater reduction in HbA1c levels in the experimental group between baseline and follow up. One study [51] evaluating individual culturally tailored care provided by NCM and CHW compared to minimal care, showed a significant decrease in HBA1c levels. The effect was significant only in the group of participants receiving a higher number of home visits (− 0.68% vs 0.43%, *p* = 0.03, *n* = 522). Another study conducted with Korean Americans immigrants [52] found that a culturally tailored program including psycho-behavioral education, home glucose monitoring with tele-transmission, and bilingual nurse telephone counseling, was associated with a greater improvement in HbA1c values (− 1.3% vs − 0.4%; *p* = 0.01, *n* = 79).

A study conducted in a rural setting [53], showed an improvement in HbA1c levels among patients exposed to diabetes education with interactive online sessions, delivered by a multidisciplinary team (0.7 ± 1.3% vs 0.1 ± 1.0%; *p* < 0.03 after adjustment for baseline HbA1c, *n* = 95).

A significant decrease of HbA1c was observed following a case management program delivered by a CHW with the support of a clinical outreach team that included home visits [19] (− 1.0% vs − 0.2%, *p* = 0.02, *n* = 233). Lujan et al. [54] tested the effectiveness of a multi-component education program led by promotoras showing a mean change of HbA1c in the intervention group significantly greater than that of the control group at 6 months (*p* < 0.001, *n* = 149).

A multicenter study [55] considered a composite outcome measure based on the achievement of target values for HbA1c, SBP, and LDL. Participants assigned to the intervention arm (health coaching group) showed higher proportions of people reaching all clinical goals (46.4% vs 34.3%, *p* = 0.02, *n* = 389) compared to usual care.

A study evaluated an education program [56] supervised by a nurse specifically trained for case management (DPP Lifestyle Program) where participants in the experimental group also received an evidence-based medication algorithm. The authors observed a significant improvement in HbA1c levels in the experimental group compared to the control (− 1.87% ± 0.81 vs − 0.54% ± 0.55; *p* = 0.011). However, no information on sample size and participant characteristics were reported.

Significant differences in blood pressure were found between groups in three studies [16, 22, 45]. A difference in means of change from baseline in diastolic blood pressure significantly favored the intervention in a multicenter study [16] where participants received intensive disease management led by practice nurse supported by link workers and a diabetes specialist (adjusted MD = − 1.91 mmHg; *p* < 0.001, *n* = 1486). In the study of Hotu et al. [22], Maori and Pacific patients with diabetes and chronic kidney diseases who received twelve months of home visits by a nurse, achieved a significant lower systolic blood pressure compared to usual care group (149 mmHg vs 140 mmHg; *p* < 0.05, *n* = 55). In a long-term follow-up study [45] (60 months, *n* = 1665), a significant reduction in SBP (MD = − 4.32 mmHg, 95% CI -6.72 to − 1.92] and DPB (MD = − 2.63 mmHg, 95% CI -3.74 to − 1.52] was detected among ethnically diverse, medically underserved patients receiving a self-management intervention with the support of home telemedicine and a nurse case manager.

Of the 14 trials reporting BMI outcome, only one [45] showed an adjusted MD of 0.40 kg/m^2^ (95% CI 0.20 to 0.60) when enhanced care through a diabetes-specialist nurse and link worker were compared to usual care.

One [56] of the two studies reporting data on weight change from baseline found a significant decrease at the end of the nine-month intervention of − 2.47 kg (±1.87) in the experimental group and + 0.88 kg (±1.84) in the control group (*p* = 0.01).

Seventeen trials assessed the impact of QI interventions on total cholesterol and/or HDL cholesterol, LDL cholesterol, and triglycerides. In three studies there were significant differences in change from baseline between groups.

At six months follow-up, Garcia et al. [57] reported statistically significant differences between the control and intervention group for total cholesterol (*p* = 0.003) and LDL cholesterol (*p* = 0.014), although not for triglycerides (*p* = 0.179).

A significant effect on total cholesterol and triglycerides was found in Kim et al. [52]. The intervention group showed significantly lower levels of total cholesterol (− 24.7 mg/dl vs 7.2 mg/dl; *p* = 0.03) and triglyceride (− 84.6 mg/dL vs − 4.2 mg/dL; *p* < 0.05) when compared with the control group. The intervention group also showed a trend toward a lower HDL, but this difference was not statistically significant (*p* = 0.059).

In Shea et al. [45], the intervention group experienced net improvement in LDL cholesterol level relative to usual care; a significant between groups difference was reported at 5 years (MD = − 3.84; 95% CI -7.77 to − 0.08).

Glucose monitoring was considered in four studies [19, 36, 46, 48]. The study conducted by McDermott et al. [19] showed that participants in the control group (waiting-list group) were more likely to self-monitor their glucose level than the experimental group.

Nine trials reported adherence to diet but measures and scores used varied between trials. Three studies found a difference between groups.

Babamoto et al. [58] found that the proportion of patients consuming two or more servings of fruits and vegetables daily increased significantly in the CHW and case management groups but not in the standard provider care group. Patients’ self-reported intake of fatty foods decreased significantly from 29 to 16% (*p* < 0.05) in the CHW group but remained unchanged in the other groups.

Cramer et al. [56] used the Dietary Questionnaire to measure eating habits and observed a significant improvement in the experimental group compared with the usual care group (*p* < 0.001). Lynch et al. [59] also observed a significant increase in the number of days following a general and specific diet among participants receiving a culturally-oriented self-management program (MD = 1.9, 95% CI 0.6 to 3.1; MD = 1.2, 95% CI 0.2 to 2.2, respectively, *n* = 61), measured by the Block Food frequency Questionnaire.

Eight trials studied physical activity using different measures, and two reported an effect following the experimental intervention. One study [59] reported results from the CHAMPS (Community Healthy Activities model for Seniors) physical activity questionnaire modified for use among African Americans. At study endpoint there was a statistically significant difference between groups (MD = 2.517 Kcal/week; *p* < 0.01).

Comparing usual care with two educational programs provided by a different case manager (CHW or NCM), Babamoto et al. [58] found a significant improvement in physical activity with an increase from 28 to 63% (*p* < 0.05) in the CHW group, and from 17 to 35% (*p* < 0.05) in the standard provider care group, without any change in the case management group.

Six studies reported data on diabetes knowledge measured by validated instruments such as the Diabetes Knowledge Questionnaire [28, 54, 58], the Spoken Knowledge in Low Literacy in Diabetes Scale [57], and the Diabetes Knowledge Test [46, 52]. A significant improvement in patient’s skills was observed in three studies [46, 54, 58].

In one out of three studies considering emergency and/or hospital admissions [51, 58, 60], there was a reduction in emergency visits from baseline to 24 months among patients receiving a culturally tailored care provided by a NCM and a CHW (RR = 0.77, 95% CI, 0.59–1.00) [60].

One study [45] investigated the effect of telemedicine compared with usual care on all cause mortality but no differences between groups were reported (HR 1.01, 95% CI 0.82, 1.24).

### Studies evaluating differential intervention effects by PROGRESS factors (*n* = 7)

Seven studies conducted sub-analyses to explore a differential intervention effects across PROGRESS-Plus factors (*n* = 7) and all were conducted in developed countries. They used a parallel study design with a follow up of 12–24 months.

Table 2 gives the details of studies and results. Females, age ≥ 50, African-Americans and those with low education showed a better improvement in glycemic control. Patient education based on low-fat dietary strategies delivered by discussion groups and supported by phone contacts, produced a greater decrease in BMI, weight, and dietary behaviors among women than men [37]. At healthcare organization level, diabetes self-management supported by CHW was associated with a greater BMI reduction and an increase in exercise frequency among participants aged ≥50. One study analyzed intervention differential effect by levels of health literacy [36]. The experimental program aimed to supply information and promote diabetes self-management skills by computer multi-media including audio/video sequences. Among low literacy subjects with poor glycemic control, the authors found a greater decrease in HbA1C in the group exposed to computer multi-media education program than in the control group (− 2.1 vs. -0.3%, *p* = 0.036). No significant difference was found among high-literacy subjects. Moreover, the multimedia users with low health literacy demonstrated gains in knowledge, self-efficacy, and perceived susceptibility to complications compared with those having higher health literacy.

**Table 2:** Evidence synthesis on differential effect analyses by PROGRESS-Plus factors

Study, country	PROGRESS-factor	Intervention type	Outcome	Method of analysis	Overall intervention effect	Differential effect
Anderson 2010 [61] USA	Spanish speaking only, education level	Patient level Number of experimental conditions: 2 (1 intervention, 1 control) Intervention: • telephonic disease management (weekly, bi-weekly, or monthly) based on: 1. brief clinical assessment 2. self-management: including diet, exercise, stress reduction, smoking cessation, readiness assessment, and development of specific self-management goals 3. medication adherence 4. glucose monitoring and review of home glucose monitoring results • educational materials Personnel involved: nurse Control group: • Usual care at Community Health Center	A1c, DBP,SBP, BMI, LDL, diet behavior (BDA); physical activity (RAPA); depression measured Patient Health questionnaire (PHQ-9)	Subroups analysis and interaction analysis	No significant differences between groups for any outcomes *Retention rate* 79% vs 64%	*A1C* Spanish speakers (yes vs no) MD = − 0.10(− 0.53, 0.33) vs 0.35(− 0.17, 0.88) Educational level: (high level vs low level) MD = 0.14(− 0.30, 0.57) vs 0.00(− 0.52, 0.52) None of the interactions was significant
Anderson-Loftin 2005 [37] USA	Gender	Patient level Number of experimental conditions: 2 (1 intervention, 1 control) Intervention: • Education in low fat dietary strategies (4 weekly classes) • 1-h peer-professional discussion groups (5 monthly) • Additional educational support by phone (weekly) • Incentives for attendance Personnel involved: nurse case manager Control group: • Usual care including a referral to a local 8-h traditional diabetes class (information on nature and complications of diabetes) • Incentives for attendance	A1c, BMI, LDL, weight, dietary fat behaviors assessed by FHQ, physical activity, psychological status	Stratification by gender	A1c No significant differences Mean weight Significant effect I: - 4 lb. C: + 4.2 lb. BMI I: − 0.81 kg/mm2 C: + 0.57 kg/mm2 MD = 1.38 kg/mm2 *p* = 0.009 Dietary behaviors (FHQ score) I: 2.5 ± 0.4 C: 2.6 ± 0.4 MD = 0.2 *p* = 0.005	Men vs women A1c No significant differences Mean weight Significant effect + 5.4 lb. vs − 1.5 lb.; MD = 6.9 lb. BMI + 2 kg/mm2 vs 0.16 kg/mm2 *p* = 0.02 Dietary behaviors Significant effect (FHQ score) − 0.24 vs − 0.17
Babamoto 2009 [58] USA	Age	Healthcare level Number of experimental conditions: 3 (2 intervention, 1 control) Intervention: • Group A, CHW program, Amigos en Salud (Friends in Health): education through individual session and monitoring services; individual sessions with participants and family member; telephone calls to participants to monitor self-management, to help participants improve their diabetes self-management skills • Group B, case management: education from two linguistically competent and culturally sensitive. Patients case management were usually seen on a monthly basis + follow-up calls. Personnel involved: bilingual, trained community health workers, nurse case manager Setting: Community, home, clinic Control group Standard Provider Care: standardized clinical care by physicians and nurse practitioners, without case management or CHW services	BMI, A1C, medication adherence, diet, physical activity, emergency department admission (ED)	Logistic regression models	Mean A1c Within group CHW = 8.6 to 7.2%; *p* < 0.05 CM =8.5 to 7.4%; *p* < 0.05 Standard care = 9.5 to 7.4%;p < 0.05 No significant differences were found between groups BMI Significantly greater decrease for the CHW group compared with the standard care group OR = 2.9 (95% CI 1.1–6.6) ED Change from baseline CHW: total visit decrease 11% Case management: total visit increase 40% Standard care: increase 15% between groups at 6-month follow-up *p* < 0.05 Diet CHW group were more likely (OR = 2.43; 95% CI =1.13–5.23) to report having two or more servings of fresh fruit per day than standard care Physical activity CHW group was more likely (OR = 2.87, 95% CI = 1.34–6.17) than standard care to report exercising three or more times per week	Patients aged≥50 were less likely to have reduced BMI at follow-up OR^a^ = 0.4 (95% CI = 0.2–0.8) Exercise frequency^b^ 3 times or more per week vs 2 times or fewer per week OR = 2.2 (95% CI = 1.1–4.1)
Brown, 2011 [63] USA	Gender	Patient level Number of experimental conditions: 2 (1 intervention, 1 control) Intervention: • Diabetes self-management education (DSME) including 8 consecutive weeks of education followed by a support group session at 3 and 6 months • Experienced NCM providing: culturally tailored diabetes self-management education; individualized health guidance and assistance with overcoming cultural and environmental barriers to improving health; guidance on locating, accessing, and navigating healthcare services; enhanced coordination of health care and communication with physicians and other healthcare providers • Random observations visits Personnel involved: bilingual NCM, nurses, dietitians, and CHWs Control Group: DSME intervention only	A1c, FBG, lipids, BP, BMI, diabetes-related knowledge, health behaviors (physical activity, dietary intake, glucose monitoring)	Interaction terms in hierarchical linear and nonlinear models to test for differential impact of treatment by gender	Over time, both the experimental and control groups showed improvements in FBG levels at three and At six months For A1c the control group had greater clinical improvements at both intervals Self-reported physical activity and fat intake Improvement for both experimental and control groups	FBG, BMI: No significant differences between gender The rate of change in A1c over time did not differ significantly by gender (coefficient^ = − 0.06, t ratio = 0.25, *p* = 0.806)
Forjuoh 2014 [64] USA	Race/ethnicity	Patient level Number of experimental conditions: 4 (3 intervention, 1 control) Intervention: • Group A. self-management through personal digit assistant (PDA). Diabetes Pilot Chronic Disease Self Management Program (CDSMP): 6 week group education program to increase self efficacy • Group B. self-management through PDA • Group C. combination of A + B Personnel involved: trained facilitator, project coordinators Setting: outpatient clinic, community Control group: usual clinical diabetes care, along with patient education materials	A1C, physical activity, BMI, BP, diet	Interaction terms in multilevel models to test for differential impact of treatment by race/ethnicity	BMI and BP: Modest reductions from baseline to 12 months of follow-up for all four groups. No significant difference for other outcomes. Self care activities: Hispanic washing feet significantly more than other racial/ethnic groups (*P* = 0.02) Retention rate: CDSMP: 85%; PDA 64%, CDSMP + PDA 64%; Control 78%	A1c Modest reductions occurred in A1c from baseline to 12 months of follow-up for all/ethnic groups. There was no significant difference in A1c change over time by race/ethnicity.
Gerber 2005 [36] USA	Health literacy	Patient level Number of experimental conditions: 2 (1 intervention, 1 control) Intervention: Education by computer multi-media including audio/video sequences (“Living Well with Diabetes”) to communicate information, provide psychosocial support and promote self-management. Subject received compensation based on computer usage. Lessons in English and Spanish. Navigation provided through a simplified interface, including forward/backward buttons for user control. Advanced features included “pop-up” supplementary text information or additional testimonials related to the concurrent screen concept Personnel involved: bilingual research assistant Setting: urban outpatient clinics Control group: simple multiple-choice quizzes on diabetes-related concepts	A1c, BMI, BP, eye exam, diabetes knowledge, self-efficacy, self-reported medical care, and perceived susceptibility to complications	Stratification by level of health literacy	No significant differences for all outcomes but perceived susceptibility to diabetes complications	Lower literacy group % change A1c − 0.21 ± 2.0 vs − 0.1% ± 1.3 MD = − 0.10 [− 0.67, 0.47] People with A1c > 9% − 2.1 vs − 0.3 (*p* = 0.036) Perceived susceptibility to complications % change score= 1.48 ± 2.7 vs 0.19 ± 2.5 (*p* = 0.016) Self-efficacy trend toward greater improvement in self-efficacy 1.51 ± 1.5 vs. 0.99 ± 1.4 (*p* = 0.113) Higher literacy % change A1c + 0.3% ± 1.6 vs. -0.5 ± 1.5 MD = 0.80 [0.22, 1.38] Perceived susceptibility to complications 0.76 ± 2.5 vs. 0.29 ± 2.4 (*p* = 0.267) Medical care Improvement over time (*p* < 0.012 for time interaction) but no effect for either lower- or higher-literacy groups
Sixta 2008 [28] USA	age	Healthcare level Number of experimental conditions: 2 (1 intervention, 1 control) Intervention: Diabetes culturally self-management education with group sessions Personnel involved: promotores in consultation with a care team Control group:Usual care delivered by provider at the clinic or to a self-care management	A1C, knowledge, beliefs	Stratified analysis by age	A1C, knowledge, beliefs	A1C No difference between groups DKQ, HBQ. No difference between groups DKQ, HBQ, and HbA1c results were significantly affected by age; Slightly negative effect on DKQ scores per year of age. Slightly negative effect on HBQ scores and HbA1c levels per year of age
West 2007 [70] USA	Race/ethnicity	Patient level Number of experimental conditions: 2 (1 intervention, 1 control) Intervention: • 42 group session of behavioral weight control program focusing on attainable and sustainable changes in dietary and physical activity habits • Motivational interviewing: 5 individual sessions lasted 45 min Personnel involved: Behaviorist, nutritionist, diabetes educator, trained clinical psychologist Setting: outpatient clinic Control group: health education sessions with focus on women’s health topics	A1C, glucose monitoring	The weight patterns over time by race were examined using a two-factor repeated measures ANOVA stratified by treatment	Weight At 6 months Means: − 4.7 ± 5.4 kg vs − 3.1 ± 3.9 kg (*p* = 0.03) Over 18 months: Means: − 3.5 ± 6.8 Kg vs − 1.7 ± 5.7Kg (*p* = 0.04) A1C Decrease in both groups (*p* < 0.0001) at 6 months but not sustained at 18 months Greater decrease in the intervention than in the control group (*p* = 0.002)	Weight at 6 months regardless treatment: African-American vs White -3 kg ± 3.9 vs. -4.5 ± 5.1 kg (*p* = 0.03) Weight at 12 months regardless treatment:: − 2.3 kg ± 4.4 vs − 4.6 ± 6.8 kg (*p* = 0.09) Weight at 18 months regardless treatment: − 1.4 kg ± 4.7 vs − 3.3 ± 7.1 kg (*p* = 0.09) For African-American experimental intervention produced greater weight loss than control group at 3 and 6 months. The benefit was not sustained after 12 months A1c African American had high A1c values regardless of treatment assignment. No interaction by race Attendance between groups was comparable.

Data are means ± SD; *I* intervention group, *C* control group, *OR* odds ratio, A1c, Glycated hemoglobin; *BMI* Body Mass Index, *LDL* low density cholesterol, *BP* blood pressure, *SBP* systolic blood pressure, *DBP* diastolic blood pressure, *MD* mean difference, *FHQ* food habit questionnaire, *PHQ-9* Patient Health Questionnaire, *DSME* Diabetes self-management education, *DKQ* diabetes knowledge questionnaire, *HBQ* Health Beliefs Questionnaire

^a^multivariate analysis adjusted for study group, gender, dietary, exercise activity; ^b^univariate analysis (did not persist after the other covariates were controlled for); ^b = regression coefficient

## Discussion

Applying an equity-oriented approach, this review identified 58 RCTs (17.786 participants) evaluating QI strategies to improve the quality of diabetes care in a primary care setting.

Forty-seven studies were from USA and evaluated interventions specifically designed to reach population subgroups mainly defined on the basis of race or ethnicity. A narrow subset of these studies (*n* = 7) considered other dimensions of disadvantage as defined by the PROGRESS framework, such as socio-economic status and place of residence.

The RCTs included in this systematic review covered a wide assortment of QI strategies, varying from patient-mediated interventions with sessions of self-management supported by healthcare professionals, to provider education and other more complex programs based on changes in healthcare organization. Twenty-nine studies considered QI interventions conducted at the patient level, three at the provider level, and twenty-six at the health care organization level.

Pooling of results and quantitative synthesis was precluded by marked heterogeneity (mainly clinical), because study population, types of interventions, outcome measures, outcome assessment tools, duration of follow-up and risk of bias varied widely between studies.

QI strategies based on patient education and self-management strategies improved HbA1c levels among racial and ethnic minority participants but heterogeneity and complexity of interventions made difficult to identify the effective components of these interventions. The evidence on the effect of patient level interventions on improving other clinical and laboratory parameters, such as blood pressure, cholesterol levels and BMI, as well as self-management behaviours is scant. Few studies explored the effectiveness of other patient level strategies, including incentives and reminders. The only study included in this review [34] testing a rewards-based incentive intervention, showed effective results.

With regard to interventions at provider level, only one study reported a significant between groups difference in HbA1c reduction while no significant impact on blood pressure or BMI was observed.

Many of the studies included in this systematic review were designed to evaluate the effectiveness of changing, expanding, or integrating the roles of healthcare professionals combined with patient education to improve diabetes care and outcomes. QI interventions based on multidisciplinary teams including trained nurses or local community health workers providing culturally competent care, were associated with a significant reduction of HbA1c values. Changes in the role of health care professionals have been shown to produce an improvement in glucose control in ethnic minority communities on ethnic minority communities showed.

As far other primary outcomes considered in this review, a significant improvement in cholesterol levels was reported while n differences were found for secondary outcome measures, except for an increase in physical activity and diabetes knowledge.

Seven studies reported data on the differential effect by at least one PROGRESS factor. We did not find evidence of a differential effect by gender and race of any intervention on HbA1c levels reduction. One study reported an improvement in glucose control among a low literacy population subgroup, exposed to a culturally competent education program delivered through multi-media tools. We found some evidence of effectiveness of QI interventions in weight loss and BMI among females and weight loss among African-Americans.

In general, the heterogeneity of baseline HbA1c values and mean age of participants can affect intervention outcomes due to the biomedical challenge of lowering HbA1c from a higher baseline value. Moreover, some studies defined a minimum A1C value as inclusion criterion possibly considering patients which may not be representative of diabetic population receiving care in a real world clinical setting. Rather than implementing minimum A1C values for participant inclusion, as many of the studies reviewed incorporated, it is important (it may be worthwhile) to maintain the integrity of studying quality improvement interventions in real-life clinical settings and therefore address differences in baseline A1C values across studies in ways other than restricting patient participant inclusion.

Another relevant issue in the evaluation of QI strategies is that the control groups received a wide range of interventions, from basic education materials, usual care, to individualized coaching from community health workers. Furthermore, in many of these studies, the control group intervention was not described in detail. This is important as the usual or routine care in different settings varies by a multitude of variables including payment system, geographic location, country, and more generally, the resources and quality of services routinely provided to patients. In addition, type and quality of usual care at a health center can impact baseline values, especially HbA1c. Moreover, biases may exist depending on previous improvement activities implemented and general commitment of medical staff and organizational leadership to reducing disparities and improving care.

The conclusions of this systematic review are largely in accord with those in a previous review on this topic among socially disadvantaged population living in industrialized countries published in 2006 [74]. The review identified 17 studies, seven trials were with low SES populations, and ten focused on etno-racial groups. The small number of studies in Glazier’s review provided limited and inconclusive evidence on intervention attributes that improved diabetes quality of care and health outcomes, underlining the potential effect of some features in reducing health disparities.

Our review provides an update and a more complete overview of the available evidence considering three specific aspects: use of PROGRESS framework to capture different socio-economic dimensions; assessment of the risk of bias of included studies; and the inclusion of studies evaluating QI strategies defined according to international classification.

Using an equity oriented approach, we identified a large number of randomized studies showing that considerable strides have been made to test interventions to address health inequities in diabetes care and outcomes. Despite the increase of the number of trials, the methodological quality resulted to be low. This finding is consistent with a previous review [75] reporting that the increase in the number of RCTs on QI strategies runs parallel to the proportion of trials having at least one domain with high risk of bias. Most included trials did not report the method of randomization and description of the allocation process. The area of the greatest potential risk of bias was the inadequate blinding of participants and outcome assessors, and poor follow up. In some of included trials the general lack of reporting of methods made it difficult to assess methodological quality and thereby judge risk of bias, independently of year of publication. The issue of small sample size extends beyond the quality of those studies included in this review. There were a number of studies, both pilot and not, that were excluded from this review because they had a sample size smaller than 50. Furthermore, since most studies were carried out in USA, their degree of external validity is uncertain. Results from these studies may be less transferrable to other countries and settings due to their being tested in a market-based health care system. It is likely that the patients’ population covered by universalistic care is more heterogeneous with regard to socio-demographic and clinical characteristics. For example, those countries with universal health care systems may have more heterogeneous patient populations in a single community. It is therefore necessary to plan trials in other countries. By the same token, interventions addressing health disparities in other countries are likely to involve groups of varying social advantage or disadvantage being served under the same health center or system. The approach to addressing inequity becomes more about reducing health disparities on a more granular level requiring tools such as health equity audit.

Although the PROGRESS framework provides a vast array of disadvantage categories, there was limited heterogeneity in the dimensions of disadvantage considered in RCTs. The most common PROGRESS factor were age and race/ethnicity, this underlines the needs of further research with a focus on other characteristics such as socioeconomic status, social capital, place of residence, occupation, education, and religion. Researchers studying populations at social disadvantage must also describe the study population and the nature of their disadvantage more specifically. This is of further importance because a lack of description or definition of a socially disadvantaged group was a common reason for study exclusion in this review and others.

There is also a clear need for more RCTs at the provider level, especially those evaluating interventions based on computerized provider reminder systems. With the widespread uptake of recognition and certification programs in primary care (e.g. medical home, diabetes recognition programs,), it is likely that audit and feedback strategies using benchmarking are common among primary care practices, but are less frequently reported for effectiveness among disadvantaged patient populations.

This research reveals an overall lack of focus on interventions that address outcomes related to adherence to guidelines where disparities are stark according to the literature. The paucity of studies measuring process of care may be a reflection of the few number of QI interventions at the provider level who, in conjunction with other members of the primary care team, are responsible for performing or referring to these services. Clinical outcomes should derived from electronic health record systems, but may not be as recurrently funded as biochemical diabetes outcomes. Process outcomes or adherence to guidelines is crucial to measure and address due to the evidence of disparities that exist on the level of clinical quality and care. It is also important to note that several studies measured diabetes “self-care” or “self-management” activities but did not report results on distinct components such as medication adherence or glucose monitoring. As these clinical outcome measures are crucial in measuring effectiveness of diabetes intervention, it is important to report on these components as distinctive measures.

We see many studies that aim to evaluate interventions to improve care and/or outcomes among a disadvantaged group, but seldom do we find studies investigating the effect of QI interventions disentangled by different levels of indicators of socio-economic position or relevant socio-demographic factors. This may because practices are not disaggregating data to identify disparities within patient populations and are therefore not initiating action to address them. It should be necessary to promote and sustain a different approach including audit activities to identify inequities in care and outcomes, and then work to address these disparities. Moreover, an “equity lens” approach should be adopted by the scientific community when identifying research priorities aimed at contrasting socioeconomic differentials. This equity-oriented approach is necessary to identify and describe the appropriate target population, to define inequalities indicators, and select process and outcome indicators useful for assessing the differential effect of an intervention.

## Conclusions

Because of the methodological differences and weaknesses that precluded meta-analytic synthesis, we can draw no strong conclusions concerning the potential benefits or harms of QI strategies to reduce inequalities in access to care for patients with diabetes in primary care. Moreover, the included studies did not allow for an analysis of the differential effects of interventions across population sub-groups.

This review highlights some QI strategies for consideration and in need of further study. Health care professionals and policy makers need the best available evidence to administer and support those interventions most likely to be effective to reduce disparities in diabetes care.

## Additional files

Additional file 1: Search strategy for PubMed. (DOCX 17 kb)

Additional file 2: **Table S1.** Inclusion and exclusion criteria (PICOS). (DOCX 14 kb)

Additional file 3: **Table S2.** Quality improvement strategies: level and description. (DOCX 13 kb)

Additional file 4: **Table S3.** Characteristics of eligible studies assessing the efficacy of QI interventions in participants with type 2 diabetes. (DOCX 71 kb)
