# Supplementary material for: Quality improvement strategies at primary care level to reduce inequalities in diabetes care: an equity-oriented systematic review
Source: BMC Endocr Disord. 2018 May 29;18:31. doi: 10.1186/s12902-018-0260-4 (PMC5975519; doi:10.1186/s12902-018-0260-4)
Supplement: Supplementary file 1 — Search strategy for PubMed. (DOCX 17 kb) [file 12902_2018_260_MOESM1_ESM.docx]

**Search strategy for PubMed**

Database: PubMed < 1 January 2005 to 31 May 2016>

**Population**

1. medically underserved[tiab] OR orphan*[tiab] OR poverty area*[tiab] OR special population*[tiab] OR minority group*[tiab] OR ethnic group*[tiab] OR immigrant*[tiab] OR socioeconomic factor*[tiab]

2. ((health[tiab] OR disease*[tiab] OR morbidit*[tiab] OR mortalit*[tiab] OR racial[tiab] OR ethnic[tiab]) AND (inequalit*[tiab] OR inequit*[tiab] OR disparit*[tiab] OR equit*[tiab] OR disadvantage*[tiab] OR gradient*[tiab]))

3. ((disadvantage*[tiab] OR "low income"[tiab] OR marginalize*[tiab] OR marginalise*[tiab] OR underserved[tiab] OR "under served"[tiab] OR rdepriv*[tiab] OR poverty[tiab] OR impoverish*[tiab] OR minority[tiab]) AND (population[tiab] OR group*[tiab] OR communit*[tiab] OR neighborhood*[tiab] OR neighbourhood*[tiab]))

4. “low pay”[tiab] OR “low paid”[tiab] OR “low income”[tiab] OR unemployed[tiab] OR depriv*[tiab] OR "financial hardship" [tiab] OR "benefit recipient" [tiab] OR "benefit recipients"[tiab] OR "social position" [tiab] OR "social class" [tiab] OR “low socioeconomic” [tiab] OR “lower socioeconomic” [tiab] OR "social status"[tiab] OR poverty [tiab] OR impoverish*[tiab]

5. “lower-SES”[tiab] OR “low-SES”[tiab] OR “low-SEP”[tiab]

6. illiterate [tiab] OR educational attainment [tiab] OR educational attainments[tiab] OR "educational level" [tiab] OR "educational status"[tiab] OR "low education"[tiab] OR "educational achievement" [tiab] OR "school leaver" [tiab] OR "school leavers"[tiab] OR “low literacy”[tiab]

7. ((elderly[tiab] OR geriatr*[tiab]) AND (inequalit*[tiab] OR inequit*[tiab] OR disparit*[tiab] OR equit*[tiab] OR disadvantage*[tiab]))

8. "Sex Factors"[Mesh] OR "geriatrics"[Mesh] OR homosexuality[Mesh] OR disabled persons[Mesh] OR "Medically Underserved Area"[Mesh] OR "Poverty Areas"[Mesh] OR "Vulnerable Populations"[Mesh] OR "Minority Groups"[Mesh] OR "Health status disparities"[Mesh] OR ethnic groups[Mesh] OR socioeconomic factors[Mesh] OR developing countries[Mesh] OR refugees[Mesh] OR social environment[Mesh]

9. "socio-economic factor"[tiab] OR "socio-economic factors"[tiab]

10. ((sex[tiab] OR gender[tiab]) AND (inequit*[tiab] OR disparit*[tiab] OR inequality*[tiab]))

11. gender-based[tiab] OR gender-related[tiab] OR gender differences[tiab] OR gender factors[tiab]

12. #1 OR #2 OR #3 OR #4 OR #5 OR #6 OR #7 OR #8 OR #9 OR #10 OR #11

**Condition**

13. (("Diabetes Mellitus, Type 2"[Mesh]) OR (MODY[tiab] OR NIDDM[tiab] OR T2DM[tiab] OR T2D[tiab])) OR ("non insulin dependent"[tiab] OR "noninsulin dependent"[tiab] OR "non insulindependent"[tiab] OR "noninsulindependent"[tiab] OR "non-insulin dependent"[tiab] OR noninsulinodependent[tiab] OR "non insulin treated"[tiab] OR "noninsulin treated"[tiab] OR "non insulintreated"[tiab] OR "noninsulintreated"[tiab] OR "non-insulin treated"[tiab] OR (("non insulin" [tiab] OR noninsulin[tiab] OR "type 2" [tiab] OR "type II" [tiab]) AND diabetes[tiab]) OR "type 2 DM" [tiab] OR "type II DM" [tiab] OR DMNID[tiab] OR "type II diabetes"[tiab] OR "type 2 diabetes" [tiab] OR "type II diabetic"[tiab] OR "type 2 diabetic"[tiab] OR "diabetes type 2" [tiab] OR "diabetes type II" [tiab] OR "diabetes mellitus type 2" [tiab] OR "diabetes mellitus type II" [tiab] OR "adult diabetes"[tiab] OR "maturity onset diabetes"[tiab] OR "late onset diabetes"[tiab] OR "stable diabetes"[tiab] OR "adult diabetic"[tiab] OR "maturity onset diabetics"[tiab] OR "late onset diabetics"[tiab] OR "stable diabetics"[tiab])

**Filter for Study design**

14. randomized controlled trial [pt] OR controlled clinical trial [pt] OR randomized [tiab] OR placebo [tiab] OR drug therapy [sh] OR randomly [tiab] OR trial [tiab] OR groups [tiab]

15. animals [mh] NOT humans [mh]

16. #12 AND #13

17. #16 AND #14

18. #17 NOT #15
