# Supplementary material for: Quality improvement strategies at primary care level to reduce inequalities in diabetes care: an equity-oriented systematic review
Source: BMC Endocr Disord. 2018 May 29;18:31. doi: 10.1186/s12902-018-0260-4 (PMC5975519; doi:10.1186/s12902-018-0260-4)
Supplement: Supplementary file 2 — Table S1. Inclusion and exclusion criteria (PICOS). (DOCX 14 kb) [file 12902_2018_260_MOESM2_ESM.docx]

Inclusion and exclusion criteria (PICOS)

| **Domain** | **Inclusion criteria** | **Exclusion criteria** |
| --- | --- | --- |
| Population | Social groups (18 years and older) with type 2 diabetes mellitus of any duration of diagnosis | Children, adolescents or gestational diabetes |
| Intervention | Single or multicomponent QI intervention aimed to reduce inequalities in diabetes care | Addressed to primary prevention of the disease |
| Comparator | usual care, no treatment, waiting list control, or alternative interventions not included in QI definition.  Usual care is likely to vary widely between settings. We defined usual care as any care that patient would receive if they have not been included in the experimental arm (i.e.,routinecare) |  |
| Outcomes | Primary outcomes:  1. clinical and laboratory parameters (change in HbA1c level, systolic and diastolic blood pressure, lipid levels, Body Mass Index - BMI) 2. process indicators or adherence to guidelines (HgA1c measure, blood pressure measure, foot exam, dilated eye exam, evidence-based patient/family education).  Secondary outcomes:   1. Measures of diabetes of Self-management behaviour (home glucose monitoring, regulation of diet and exercise, diabetes knowledge, confidence and skills to control diabetes adherence to medications, attendance at office appointments when needed or as scheduled by the provider); 2. clinical outcomes (diabetes complications, avoidable hospital admissions, emergency admission and death). |  |
| Setting | Primary care | Hospital/emergency care setting or at a specialty clinic |
| Study | Randomised controlled trials (cluster or individual) with a control group | Trials including less than 50 participants |
