# Supplementary material for: Quality improvement strategies at primary care level to reduce inequalities in diabetes care: an equity-oriented systematic review
Source: BMC Endocr Disord. 2018 May 29;18:31. doi: 10.1186/s12902-018-0260-4 (PMC5975519; doi:10.1186/s12902-018-0260-4)
Supplement: Supplementary file 3 — Table S2. Quality improvement strategies: level and description. (DOCX 13 kb) [file 12902_2018_260_MOESM3_ESM.docx]

Table 2. Quality improvement strategies: level and description

| **QI strategy** | **Level** | **Description and sub strategies** |
| --- | --- | --- |
| **Provider reminder systems** | Provider | Any patient- or clinical encounter-specific information to prompt a clinician to recall information or consideration of a specific process of care.  *Computerized reminders, decision support* |
| **Provider education** | Provider | Interventions including educational workshops, meetings, lectures, and educational outreach visits.  *Cultural competency training, motivational interviewing training* |
| **Audit and feedback** | Provider, health care systems | Any summary of clinical performance for health care providers or institutions.  *Benchmarking, quality indicators and reports* |
| **Facilitated relay of clinical data to providers** | Provider | Transfer of clinical information collected from patients and relayed to the provider.  *Telemedicine, mobile phone software* |
| **Patient education** | Patient | Introduction of print or audio-visual materials in an in-person individual or group setting.  *Classes, pamphlets, intensive education strategies including self-management* |
| **Promotion of self-management** | Patient | Distribution of materials or access to resources that enhances the patients’ ability to manage their condition, the communication of clinical data, or communication with the health care team.  *Materials or devices promoting self-management* |
| **Patient reminders** | Patient | Any effort directed by providers towards patients that encourages them to keep appointments or adhere to other aspects of the self-management of their condition.  *Phone calls to patients, secure messaging to patients* |
| **Organizational change** | Health care systems | Any intervention that includes at least one of the following features: disease or case management, team or personnel changes, communications or case discussions, Total Quality Management or Continuous Improvement, changes in medical record systems.  *Case Management, multi-disciplinary teams, increasing staff* |
| **Financial, regulatory or legislative incentives** | Health care systems, provider, patient | Any intervention having features consistent with at least one of the following: financial incentives directed at providers or patients, system-wide changes in reimbursement, changes to provider licensure or institutional accreditation requirements.  *Financial incentives for providers based on achievement of performance goals, patient co-payments for certain visit types* |
