# Supplementary material for: Quality improvement strategies at primary care level to reduce inequalities in diabetes care: an equity-oriented systematic review
Source: BMC Endocr Disord. 2018 May 29;18:31. doi: 10.1186/s12902-018-0260-4 (PMC5975519; doi:10.1186/s12902-018-0260-4)
Supplement: Supplementary file 4 — Table S3. Characteristics of eligible studies assessing the efficacy of QI interventions in participants with type 2 diabetes. (DOCX 71 kb) [file 12902_2018_260_MOESM4_ESM.docx]

Characteristics of eligible studies assessing the efficacy of QI improvement strategies in participants with type 2 diabetes

Patient level (n=29)

| Study,  Country | Population targeted | Longest Follow up (months) | Experimental intervention  Strategy  description | Control intervention | Outcomes | Main results  (experimental versus control) |
| --- | --- | --- | --- | --- | --- | --- |
| Anderson 2010[61] Connecticut, USA | Multi ethnic: hispanic/African Americans/Latinos  Mean age:  Women:58%  64% Puerto Rican | 12 months | Disease management (n=146) by telephone by nurse | Usual care (n=149): education + self-management | HbA1c, DBP,SBP, BMI, LDL, diet behavior (BDA); physical activity (RAPA); depression measured Patient Health questionnaire (PHQ-9) | No significant differences between groups for any outcomes  Retention rate: 79% vs 64% |
| Anderson-Loftin 2005[37] South Carolina,  USA | African Americans  Mean age: 57.3  Women 59% | 6 months | Patient education program (n=49), weekly group classes by a nurse case manager with nutrition focus, support groups, and weekly telephone follow-up | Usual care (n=48), referral to a local 8-hour traditional diabetes class. | HbA1c, BMI, cholesterol, weight, Dietary behavior measured by FHQ; physical activity | *HbA1c*  No significant differences between group  *BMI*  Significant reduction in intervention  Means: intervention -0.81 kg/m^2^ vs control group (mean+0,57 kg/m^2^ (P=0.009)  *Cholesterol*  No significant differences between group  Gender:  *BMI*  Significant increase in men  Means: men +2 kg/m^2^ vs women + 0.16 kg/m^2^ (p=0.02)  *Weight*  -1.8 kg vs +1.9 kg  *Gender*  Significant increase in men vs women  +5.4 lb vs -1.5 lb;  *Self-reported Dietary habits*  MD=0.2 points (P=0.005)  *Men vs women*  Significant increase in men (mean = –0.24) vs women (mean –0.17) (P<0.05)  *Retention rate*  78% vs 56% |

| Study,  Country | Population targeted | Longest Follow up (months) | Experimental intervention  Strategy  description | Control intervention | Outcomes | Main results  (experimental versus control) |
| --- | --- | --- | --- | --- | --- | --- |
| Baradan 2006[62] Glasgow, UK | South Asians  Mean age: 58.4  Women: 48%  Mean Years of Education: 6.6 | 3 | Patient education (n=59): 3 group sessions with didactic elements (lecture) and interactive group discussion. Support material (visual aids, food examples), booklets and leaflets | Usual care (n=59) | Self-reported patient's knowledge, attitude, practice about diabetes | Increase in scores significantly higher in the intervention but  No significant difference between groups.  Improvement in all outcomes in both ethnic control and white control group  Retention rate: 25.5% vs 39% |
| Berry 2016[17]  North Carolina,  USA  Cluster RCT | Low-income  mean age: 51.4 years  Women:89.3%  77.4% Non Hispanic-black | 15 | Patient Education (n=40), 5 visit sessions led by an interdisciplinary group (physician and nurse practitioner) and one individualized session | 5 individual education sessions (n=40) with physician or nurse practitioner | BP, HbA1c, glucose monitoring, physical activity, medication adherence, hospitalization, diet measured by Stanford diabetes self-management questionnaire | Mean HbA1c  Reduction in the intervention more than the control group  Mean 7.6% vs 9.3% (p=0.001)  HDL  Significant increase in the intervention group:  50.3 ± 12.5 mg/dl vs 43.3 ± 12.1 mg/dl (p=0.033).  Triglycerides  Significant reduction in the intervention group: 115.2 mg/dl ± 59.1 vs control group 172.0 mg/dl ± 12.1 (p=0.02)  LDL and BP: NS  Improvement in patient' satisfaction in the intervention group (p=0.001)  Heart rate  Significant reduction in intervention 75.3 vs control group 81.3 (p=0.031)  Retention rate:88% vs 83%. |

| Study,  Country | Population targeted | Longest Follow up (months) | Experimental intervention  Strategy  description | Control intervention | Outcomes | Main results  (experimental versus control) |
| --- | --- | --- | --- | --- | --- | --- |
| Brown 2005 [35]Texas, USA | Latinos  Mean age: 49.6 years  Women: 60.1% | 12 | Self-management patient education compressed (n=114): 22 contact  hours over 12 months, group education sessions led by a multi-disciplinary care team | Extended education: 52 contact hours over  12 months, group sessions (n=102) | HbA1c, BP, BMI, cholesterol, diabetes knowledge | No significant differences between groups for any outcomes.  People attended ≥50% of the intervention:  HbA1c %change= −0.6 for the “compressed” and −1.7% for the “extended group (p<.001) |
| Brown 2011*[63]  Texas-Mexico,  USA  Cluster RCT | Mexican Americans  Mean age:49,4 years  Women:69% | 6 | Self-management Patient Education and interactive support group session (n=48) delivered by dieticians and CHW + individualized follow-up care delivered by a nurse case manager | self-management education only (n=35) | HbA1c, FBG, lipids, BP, BMI, diabetes-related knowledge, health behaviors (physical activity, dietary intake, glucose monitoring | HbA1c: NS  Participants who attended ≥50% of intervention sessions  resulted in greater reductions in HbA1c, larger in the control group.  FBG: NS.  BMI: NS.  BP:NR  Improvements in knowledge, diet and physical activity, and clinical outcome in both groups. |
| Davis 2010[33]  South Carolina,  USA | African Americans  Mean age: 59.6 years  Women:74.6%  73.9% African Americans | 12 months | Self-management education via videoconferences (telehealth n=85): 3 individual and 10 group sessions | Usual care + brief individual education session control (n=80) | HbA1c, LDL, BP, BMI, albumin-to-creatinine ratio, self-report eye exam | HbA1c  Improvements at 12 months was significantly greater in the intervention group compared with usual care  8.2% ± 0.4 vs 8.6% ±0.3 (p=0.004)  LDL  89.7 ±6.9 mg/dl vs 103.1 ± 6.8 mg/dl (p=0.02)  No difference in other clinical measures.  % of participants reported receiving an eye exam: 81.2% vs 38.8% (p=0.0001)  Retention rates at 6 and 12 months were 90.9 and 82.4%, respectively. |

| Study,  Country | Population targeted | Longest Follow up (months) | Experimental intervention  Strategy  description | Control intervention | Outcomes | Main results  (experimental versus control) |
| --- | --- | --- | --- | --- | --- | --- |
| Forjuoh 2014 [64]  Texas,  USA | Racially/ethnically diverse  Mean age:57.6 ± 10.9  Women: 55.2%  Non-Hispanic white 60.1%  Non-Hispanic black 16.2%  Hispanic 20.2%  Other 3.5% | 24 | Group A. CDSMP self-management through group classes, self- care software (n=101) led by a trained facilitator;  group B. PDA diabetes self-care software (n=81); group C. combination of CDSMP+PDA (n=99) | Usual care (n=95) + publicly available Texas Diabetes Council patient education materials. | HbA1c, BMI, BP, diet, physical activity measured by the Summary of diabetes self-care activities | HbA1c  Control =-0.7%  CDSMP=1.1%,  PDA= -0.7%  CDSMP + PDA=-1.1%  No significant difference between groups (p = 0.771).  BMI and BP: Modest reductions from baseline to 12 months of follow-up for all four groups.  No significant difference for other outcomes.  Subgroup analysis:  No significant differences by race  Self care activities  Hispanic washing feet significantly more than other racial/ethnic groups (P=0.02)  Retention rate:  CDSMP: 85%; PDA 64%, CDSMP + PDA 64%; Control 78% |
| Frosch 2011[25]  Los Angeles,  USA | Low-income; racially/ethnically diverse: African American, Latino  Mean age:55.5  48.5% women  55.8% Latino | 6 | Self-management support education (n=100): video education and telephone coaching from a bilingual diabetes nurse, education materials | Education (n=101): 20-page brochure "4 Steps to Control Your Life | HbA1c, LDL, BP, BMI, diet, physical activity, glucose monitoring | Reduction of clinical measures in both groups but no significant difference between groups  Retention rate: 100% vs 72% |

| Study,  Country | Population targeted | Longest Follow up (months) | Experimental intervention  Strategy  description | Control intervention | Outcomes | Main results  (experimental versus control) |
| --- | --- | --- | --- | --- | --- | --- |
| Gerber 2005[36] Chicago,  USA | Low health literacy  mean age:54.5 years  women: 66.5%  female: 70%  low health literacy: 56% | 12 | Self-management education (n=122) by computer multi-media application in clinic waiting rooms to provide information, psychosocial support and self-management to patients | Usual care (n=122) | Self-reported HbA1c, BMI, BP, eye exam, immunization in the previous year, perceived susceptibility, self-efficacy  Laboratory measures | No significant differences for all outcomes but perceived susceptibility to diabetes complications;  Subgroup analysis  High health literacy  Mean HbA1c%  –0.5% vs 0.3% (p= 0.043);  Low literacy with Hb>9%  -2.1 vs -0.3 (p=0.036)  Other outcomes: No significant differences between groups  Self-report in perceived susceptibility to complications: 1.19 vs. 0.24 (p= 0.009)  Retention rate: 77% vs 72% |
| Gregg 2007[65] San Francisco,  USA | Low-income, Hispanic  Mean age: 50.9 years  Women:47% | 3 | Self-management education+ acceptance and commitment therapy (ACT) (n=43) led by research personnel | Education alone (n=38) | HbA1c, BMI, self-reported physical activity, diet, glucose monitoring; satisfaction with treatment; acceptance of diabetes-related thoughts measured by AADQ | HbA1c:NS  Improvement in experimental group (p=0.009)  Glucose level:NS  Self-management outcomes:  significant effect for ACT over education alone (p=0.043)  Acceptance score:  significant and large effect for ACT group (p=0.011)  Retention rate: 93% vs 87% |

| Study,  Country | Population targeted | Longest Follow up (months) | Experimental intervention  Strategy  description | Control intervention | Outcomes | Main results  (experimental versus control) |
| --- | --- | --- | --- | --- | --- | --- |
| Gucciardi 2007[20] Toronto,  Canada | Portugese-speaking minority  Mean age:59.7 years  Women:68.7% | 3 | Individual counseling + group education led by multi-disciplinary care team (n=41) | Individual self-management education by educators (n=46) | HbA1c, adherence to nutrition management measured by Summary of Diabetes Self-care Activities  Questionnaire (SDCA) | HbA1c  Improvement in the overall cohort at follow up time (p<0.01) but no differences between groups.  Significant differences between groups for perceived behavioral control, intentions towards, and nutrition adherence  (p<0.05)  Retention rate: 61% vs 78% |
| Guo 2014[21]  China | Low socioeconomic  Mean age:58 years  Women:56.1%  <high school education: 83.3% | 6 | Group A: Education+glucometer+incentives if goal (n=43); Group B: Education+glucometer+test strips at no incentives (n=41) | Education + no equipment (n=48) | HbA1c, BMI, cholesterol, physical activity, diet, medication adherence, glucose monitoring | HbA1c  A vs C  7.29% ±0.58 vs 7.73% ±0.57 (p<0.05)  All groups had decreases in HbA1c but group A had the largest reduction, -0.97% compared to -0.62 and -0.57, respectively, for the no cost and control groups;  Group A had decrease in medical costs whereas other group did not (-159 yuan)  Retention rate  93% vs 90% vs 85% |
| Heisler 2014[66] Detroit,  USA | Low-income + racially/ethnically diverse: Latino, African American  Mean age 51.5 years  Women: 71%  Hispanic: 57%  not working: % 66 | 3 | Self-management education (n=93): individual sessions with tablet aid tool by a CHW (iDECIDE) | Education materials and individual education sessions (n=95) delivered by CHW | HbA1c, self-reported diabetes care self efficacy, diabetes distress and medication adherence | Improvements in both groups for all outcomes.  Differences between experimental group compared with the print materials group for:  satisfaction with helpfulness of medication information (p<0.028);  clarity of medication  information (p<0.007);  distress (p<0.001)  Retention rate: 94% vs 94% |

| Study,  Country | Population targeted | Longest Follow up (months) | Experimental intervention  Strategy  description | Control intervention | Outcomes | Main results  (experimental versus control) |
| --- | --- | --- | --- | --- | --- | --- |
| Hill-Briggs[29] 2011 Baltimore,  USA | Low-income  Mean age: 61.3±10.9  Women: 58.9%  Living in poverty: 57.1% | 3 months | Self management education (n=29): Intensive training group and CVD education and 8 problem-solving training sessions (Project DECIDE) | Condensed (n=27): one diabetes and DVD education session and one problem-solving training session | HbA1c, BP, cholesterol, medication adherence, glucose monitoring, diet, physical activity | HbA1c  MD= -0.72% (−1.42, -0.01)  (p=0.02) in favor of the intensive intervention (Univariate model)  People with suboptimal baseline DBP  Median reduction=−7.17mmHg  People with suboptimal baseline SBP  Median reduction=−14.67 mmHg  Improvements in both groups in knowledge (p<0.001), problem-solving (p<0-001), and self-management behaviors (p=0-04)  Retention rate: 93.1% vs 96.3% |
| Khan 2011[26] Chicago,  USA | Racially/ethnically diverse: Hispanic, African American, Asian minorities  Mean age: 52.4 years  Women 43% | 3 months | Self management education in waiting area (n=53) by computerized educational self-management program | educational materials (n=47) | HbA1c, BP, BMI, physical activity, diet, glucose monitoring, medication adherence, diabetes knowledge | HbA1c  7.6±1.8 vs 8.2±2.5 (p=0.006)  No difference between groups for BMI and blood pressure  Number of diabetes medications  Increase in the experimental group after 3 months (p=0.017)  Self-reported physical activity:  Improvement in the control group compared with the multimedia group (0.9 days/week vs. 0.1, p=0.017). |
|  |  |  |  |  |  | Retention rate: 76% vs 73% |
| Levy 2015[55]  New York,  USA | Low socio-economic status  Mean age: 46.7 years  Women=51%  Income, not response=44% | 3 | Mobile Insulin Titration Intervention-MITI (n=33): Web-based health management for patient monitoring | Standard clinic care (n=28) | N° patients in the intervention arm reached their optimal insulin glargine dose, HbA1c, adverse outcomes, satisfaction outcomes | Increase in optimal insulin glargine dose  88% vs 37% (p<.001).  HbA1c: NS  Missing data was a limitation in examining change in HbA1c  Adverse effects:  5 cases of hypoglycemia; 3 patients in the MITI arm and 2 in the usual care arm  Retention rate: 85% vs 50% |

| Study,  Country | Population targeted | Longest Follow up (months) | Experimental intervention  Strategy  description | Control intervention | Outcomes | Main results  (experimental versus control) |
| --- | --- | --- | --- | --- | --- | --- |
| Negarandeh 2013 [41]  Iran | Low health literacy  Mean age: 51.4 years  Women: 42.5%  Educational: 79.6% | 6 | Group A  Education via illustrated contents (Pictorial) (n=45): education (3 weekly sessions);  Group B  Education via Teach back (n=45): education based on teach back strategy (3 weekly sessions) | Group C  Standard care education (Education material+ visit by endocrinologist + support to patients) (n=40) | Knowledge, medication and diet adherence | Significant differences in the diabetes-specific knowledge to self-management, and adherence to dietary regimen (p < 0/05).  Knowledge  A vs C  MD=-5.24 (SE=0.54) (95%IC:-6.54;- 3.93)  B vs C  MD=5.91 (SE=0.54) (95%IC:-7.21;- 4.61)  Medication adherence  A vs C  MD=−2.40 (SE=0.30) (95%CI: -3.12;- 1.69)  B vs C  MD=-2.71 (SE=0.30) (95%CI:- 3.43;-1.99)  Dietary adherence  A vs C  MD=-2.24 (SE=0.17) (95%CI:- 2.67;-1.81)  B vs C  MD=-2.52 (SE=0.18) (95%:-2.95;-2.09)  Retention rate: 88.9% vs 97.7% vs 89% |

| Study,  Country | Population targeted | Longest Follow up (months) | Experimental intervention  Strategy  description | Control intervention | Outcomes | Main results  (experimental versus control) |
| --- | --- | --- | --- | --- | --- | --- |
| Philis  Tsimikas 2011[34]  San Diego,  USA | Mexican-Americans  Mean age: 50.7 years  Women = 71.5%  Mexican:87.5 | 10 | Culturally sensitive self-management education (n=104) PROJECT DULCE: group classes delivered by a trained peer educator | usual care (n=103)+ a glucose monitor | HbA1c, LDL, BP, BMI | At 10 months  Improvements in experimental group  Within-group analyses:  HbA1c mean change from baseline= -1.5% (p<0.01)  Total cholesterol: -7.2 mg/dL (p< 0.05)  HDL: +1.6 mg/dL (p<0.05)  LDL: -8.1 mg/dL (p< 0.05)  BMI and BP: no change  No significant changes in the control group.  No differences between groups  Retention rate  66.5% vs 84.5% |
| Rosal 2011[27] Massachusetts,  USA | Low-income Latinos  >65 years: 53%  Women:76.6%  Self-reported household income  10,000/year: 55.3%  Unemployed/looking for a job=61.7% | 12 | Self-management education (n=124): educational group and individuals sessions led by nutritionist/health educator + glucose meter + counseling | Usual care (n=128) | HbA1c, diet, physical activity, BP, BMI, glucose monitoring | Greater session  attendance significantly associated with lower HbA1c outcome at 12 months, p= 0.005  Between groups differences  HbA1c, at 4 months  MD=-0.53 [-0.92 to -0.14] (p>0.008)  HbA1c, at 12 months: n.s.  Alternative Healthy Eating Index (measure of dietary quality)  MD=2.83, p=0.014  % of saturated fatty acids intake  MD=22.68, p=0.003  Diabetes knowledge  MD=0.056, p=0.001 Self-efficacy  MD=0.235, p=0.001 Retention rate: 100% |

| Study,  Country | Population targeted | Longest Follow up (months) | Experimental intervention  Strategy  description | Control intervention | Outcomes | Main results  (experimental versus control) |
| --- | --- | --- | --- | --- | --- | --- |
| Schillinger 2009 [67]  San Francisco,  USA | Ethnically diverse, low-income | 12 | Group A  Interactive weekly automated telephone self-management  support with nurse follow-up (ATSM) (n=112)  Group B  Monthly group medical visits with  physician and health educator facilitation (GMV) (n=113) | Usual care (n=114) | HbA1c, BP, BMI, diet, physical activity, medication adherence, glucose monitoring | ATSM vs control  Glycemic control, BMI: n.s.  Diabetes self-efficacy :n.s.  SMD=0.41, p=0.003  Interpersonal communication:  MD=0.34, p=0.03  Self-management  Functional outcomes (bed stays)  MD=0.4, p=0.004  Quality of life ( SF-12 mental health)  MD=0.31, p=0.03  Retention rate  90% vs 88% vs 92% |
| Shahid 2015 [24]  Pakistan | Rural setting, lack access to medical services  Intervention group  mean age:49 years  Women:39% | 4 | Self-management Blood Glucose (n=220) : glucometer+ regular feedback by phone + education material | Advice regarding their medications, diet, lifestyle changes, care and SMBG levels by the physician, nutritionist and diabetes educator (n=220) | HbA1c, BMI, BP, LDL, physical activity, diet, medication adherence, glucose monitoring | Reduction (p < 0.001) in  hypertension, BMI, mean LDL levels and mean HbA1c levels in both groups  No differences between groups  Improvements in diet for intervention group  (p < 0.001) in following diet plan from 17.3% at baseline to 43.6% at endline while in the control group there was an insignificant increase (p=0.522) from 13.6% at baseline to 15.9% at endline.  Physically active  increase in patients from 16.4% to 44.5% whereas an insignificant improvement was observed in control group from 14.1% to 16.4%  Retention rate:100% |

| Study,  Country | Population targeted | Longest Follow up (months) | Experimental intervention  Strategy  description | Control intervention | Outcomes | Main results  (experimental versus control) |
| --- | --- | --- | --- | --- | --- | --- |
| Skelly 2009 [68]  North Carolina,  USA | African American rural setting  Mean age=67 years  Women: 100% | 9 | Group A  Self management: home-based, nurse-delivered symptom-focused education and counseling (n=60)  Group B  A + booster telephone calls (n=55) | Attentional group=skills training for weight management and diets (n=59) | HbA1c,  Self-care; symptom distress;  quality of life | HbA1c declined significantly in the whole sample (0.57%) with no differences between study arms. Participants in the booster arm decreased HbA1c by 0.76%.  Symptom distress, perceived quality of life, impact of diabetes, and self-care activities improved significantly for the whole sample with no significant  differences between study arms.  Retention rate:97% vs 98% |
| Tang 2015 [38]  Michigan,  USA | African American  Mean age:56.3 years  Women: 67%  Race: African American 100% | 3 | Diabetes self-management education (DSME) program + 12-month peer support intervention (n=54) | DSME program (n=52) | HbA1c, cholesterol, BP, BMI , social support, distress | HbA1c  No difference between groups  LDL  MD=-15 mg/dL vs  (95%CI=-28.5; -1.5) p =0 .03;  SBP  MD=-10 mm Hg, (p=0 .01)  DBP  MD=-8.3 mm Hg, (p =0 .001)  BMI  MD=-0.8 kg/m^2^, (p = 0.032)  Pyschosocial measures  No difference between groups  Retention rate: 63% vs 62% |

| Study,  Country | Population targeted | Longest Follow up (months) | Experimental intervention  Strategy  description | Control intervention | Outcomes | Main results  (experimental versus control) |
| --- | --- | --- | --- | --- | --- | --- |
| Toobert 2011[39] Colorado,  USA | Latinas women  Mean age: 57.15 years  Women: 100% | 24 | Education (n=142): group classes to promote a culturally adapted Mediterranean lifestyle, ¡Viva Bien! Project | Usual care (n=138): management of complications associated with diabetes, monitoring of health factors, compliance with the ADA guidelines | HbA1c, diet, BMI, weight, physical activity, measures of problem-solving and self-efficacy | At 6 months  Improvements on measures of problem solving, self-efficacy, and perceived supportive resources  At 24 months  Improvement in experimental vs control group in fat intake, psychosocial outcome  BMI  35 kg/m2 vs 32 kg/m2; MD=0.40 (p<0.05)  Other outcomes: no difference between groups  Retention rate  61% vs 85% |
| Walker 2011[32]  New York,  USA | Low-income, ethnic minorities (black, Hispanic), urban  Mean age:55.5 ±7.3  Women 67.1%  Hispanic:61.6% | 12 | Self-management (n=262): 10 self-management telephone calls by health educator | Information material by email (n=264) | HbA1c, diet, physical activity, medication adherence for OGLA (insulin use | HbA1c  Significant reduction  -0.23±0.11% vs 0.13±0.13% (p< 0.04)  Adjusting for baseline HbA1c, sex, age, and insulin use  MD=0.40% (95% CI 0.10–0.70, P=0.009)  Medication adherence  was associated with change in HbA1c (p < 0.01);  improvement in medication adherence was associated (p= 0.005) with experimental intervention, but only among those not taking insulin  Retention rate:87% vs 81% |

| Study,  Country | Population targeted | Longest Follow up (months) | Experimental intervention  Strategy  description | Control intervention | Outcomes | Main results  (experimental versus control) |
| --- | --- | --- | --- | --- | --- | --- |
| Wayne 2015[69] Toronto,  Canada | Low socio-economic status  Mean age:53.2 years  Women: 72%  Unemployed: 36%  $0-$9999: 22% | 6 | Self-management education (n=67): monitoring health data by mobile phone and software +health coaching (n=67) | Health coaching only (n=64) | HbA1c, BMI, psychometric assessments measures by validate scale | No significant difference between group both for all outcomes  Retention rate: 72% vs 77% |
| Weinstein 2014[42]  New York,  USA | Low-income, Latinos  Mean age:55.5 years  Women: 69%  Latino: 48.7% | 3 | Self management education+ incentives (n=45): group education session on benefits of fruit and vegetable consumption from a trained physician and/or medical student, | Standard of care (n=34): visits + education by educator and/or dietician | Self-report dietary habits, HbA1c, LDL, HDL, total cholesterol, BP | Increase in the number of participants in the intervention arm who reported purchasing from a farmers market;  At 12 weeks  81% vs 48% reported ever purchasing from the market (P = .003)  55% of participants reported difficulty affording fresh fruits and vegetables (P = .008). No difference between arms  Clinical measures: no difference between groups  Retention rate: 100% |
| West 2007 [70]  Alabama,  USA | African American women  mean age:53 years  women:100%  African American: 38%  BMI≥30 | 18 | Motivational interviewing (n=198): individual motivational interviewing sessions + group-based behavioral obesity treatment | individual health education sessions (n=108) delivered by master health educators | Weight, HbA1c, glucose monitoring | Weight  At 6 months  Means: -4.7±5.4 kg vs -3.1±3.9 kg (p=0.03)  Over 18 months:  Means: -3.5±6.8 Kg vs -1.7±5.7Kg (p=0.04)  Subgroup analysis  African-American lost less weight than white women  Glycemic control  Improvement in both conditions (p<0.0001) during the intervention period but were not maintained during follow-up  HbA1c  Decrease in the experimental group but no difference between groups  Subgroup analysis  African-American had higher HbA1c values each measurement time in both groups  Retention rate:94% vs 92% |

Characteristics of eligible studies. Provider level (n=3)

| Study,  Country | Population targeted | Longest Follow up (months) | Experimental intervention  Strategy  description | Control intervention | Outcomes | Main results  (experimental versus control) |
| --- | --- | --- | --- | --- | --- | --- |
| Phillips 2005[43] Georgia,  USA | African Americans, low-income  Mean age: 59 years  Women:67%  African American: 94% | 36 | Group A. only reminders (computerized, n=1943); Group B. Feedback only (n=1049). Group C. Reminders and feedback (n=1063). | No intervention (n=983) | HbA1C, BP, LDL | HbA1c  Improvement in feedback+reminder vs control group:  0.6% vs 0.2%(P<0.02)  Attainment of the A1C  goal was  facilitated by greater age, more diabetes cinic visits, longer follow-up,  and the feedback intervention.  SBP  Improvement in feedback reminder (- 3.4 mmHg) and feedback only (-3.2 mmHg) but no difference between groups  Attainment of the sBP goal was facilitated by having had more diabetes clinic visits, longer follow-up, and the feedback intervention.  LDL  Improvement for all intervention arms, the change was greatest with the feedback reminders arm (-18 mg/dl)  Retention rate:n.a. |

| Study,  Country | Population targeted | Longest Follow up (months) | Experimental intervention  Strategy  description | Control intervention | Outcomes | Main results  (experimental versus control) |
| --- | --- | --- | --- | --- | --- | --- |
| Seligman 2005[71] California,  USA | Low health literacy  Mean age: 62,8 years  Women:42,3%  53% Hispanic  Inadequate Health literacy: 74% | 1 week | Reminder system (n=95): physicians received notification of patients' health literacy level | No feedback of patient's health literacy level (n=87) | HbA1C, physician management strategies, visit satisfaction and perceived effectiveness | Intervention physicians were more likely to use >3 recommended strategies during the visit (21% vs 8%, OR=3.2);  Satisfaction:  physicians in the intervention group were less satisfied with the visit (81% vs 93% OR=0.3). change % in HbA1c (mean)  Improvement in the intervention group but no difference between groups  MD=- 0.27% (95%CI -0.80 to -0.27, p=0.26)  Retention rate: 95% vs 97% |
| Welch 2015 [44]  Massachussets,  USA | Latino, low-income, urban  Mean age:55 years  Women: 60%  Hispanic: 100% | 6 | Reminder system for decision support (n=199): internet-based diabetes dashboard used by the care team for 6 months + diabetes education visits by a diabetes nurse or dietician | Usual diabetes care (n=200): individual patient visits + education by care team | %patients achieving HbA1C<7%, HBA1c, BP, BMI, diabetes distress, social distress | %patients achieving glucose control  15.8% vs 7%  HbA1c  MD=-0.80 [95%CI-0.82, -0.78]  BMI and BP  No significant difference between groups  Lower diabetes distress and social distress for intervention group  Retention rate: 90.5% vs 86.4% |

Characteristics of eligible studies. Health care system level (n=)

| Study,  Country | Population targeted | Longest Follow up (months) | Experimental intervention  Strategy  description | Control intervention | Outcomes | Main results  (experimental versus control) |
| --- | --- | --- | --- | --- | --- | --- |
| Babamoto 2009 [58]  Los Angeles,  USA | 318 Hispanic/Latino  Women:64%  the mean age: 50 years  (range = 18-87 years); and | 6 | Group A (n=75)=10 weeks individual educational sessions by Community health workers (CHW)+culturally appropriate education materials delivered by bilingual (Amigos en Salud); Group B. case management (CM) (n=60): diabetes care +education delivere by nurse case manager | usual care care (n=54): routine visits by physicians and nurse, education material | BMI, A1C, medication adherence, knowledge, diet, physical activity, Emergency admissions | mean HbA1c  Within group  CHW = 8.6% to 7.2%; p < 0.05  CM =8.5% to 7.4%; p <0.05  Standard care=9.5% to 7.4%;p < 0.05  No significant differences were found between groups  BMI  No significant difference between groups  ED  Change from baseline CHW grp: total visit decrease 11%  Care management grp: total visit increase 40% Control grp: increase 15%†  Significant improvements in health status, medication adherence, dietary habits, physical activity compared to control.  Diet  CHW group were more likely (OR = 2.43; 95% CI =1.13-5.23) to report having two or  more servings of fresh fruit per day than standard care  Physical activity  CHW group were more likely (OR = 2.87, 95% CI = 1.34-6.17) than standard care to report exercising three or more times per week.  Mean Diabetes Knowledge Scale score  14.7 vs 11.0 vs 10.6  Logistic regression models for HBA1c and BMI Age:  50 and older OR= 0.4, 95% CI 0.2-0.8  Multivariate analysis:  CHW group associated with BMI decrease  OR= 2.9 (95% CI 1.1-6.6)  Older age associated with decreased BMI OR= 0.4, 95% CI = 0.2-0.8 ) (controlled for study group, gender, and dietary and exercise habits).  Association between exercise frequency and decreased BMI in the univariate model (OR = 2.2, 95% CI = 1.1-4.1); however, this association did not persist after the other covariates were controlled for.  Retention rate  72% vs 57% vs 50% |

| Study,  Country | | Population targeted | | Longest Follow up (months) | | Experimental intervention  Strategy  description | | Control intervention | | Outcomes | | Main results  (experimental versus control) | |
| --- | --- | --- | --- | --- | --- | --- | --- | --- | --- | --- | --- | --- | --- |
| Bellary 2008*[16]  21 inner centre,  UK | | South Asian ethnic background  Women=48%  56% range 45-64  years | | 24 | | Enhanced care (n=868): diabetes care with additional practice nurse, link worker and diabetes-specialist nurse | | usual care (n=618) delivered by nurse | | HbA1C, BP, MAP, cholesterol, BMI, waist, LAB measures, CHD risk (Framingham CVD score) | | HbA1c  Crude MD=-0.18 (p<0.0037)  Adjusted MD=ns  DBP  Crude MD=-1.6 mmHg (p<0.007)  Adj MD=-1.91 mmHg (p<0.001)  mean arterial blood pressure  Adj MD=-1.4 (p<0.018).  BMI  Crude MD=0.38 (p<0.001  Adj MD=0.40 kgm2 (0.20 to 0.60)*p<0.001  Adjusted for confounding and clustering  other outcomes: ns  Retention rate: 86% vs 86% | |
| Cramer 2007[56]  New York, USA | | NR | | 9 | | Patient education program (DPP Lifestyle  Program) delivered by a nurse receiving extensive supervised training for case manager + evidence-based medication algorithm | | usual care: patient education with no case management | | HbA1c, weight, physical activity, diet, diabetes medication | | HbA1c change  -1.87%(±0.81) vs - 0.54% (±0.55) (P = 0.011)  Weight change  –2.47 (±1.87) kg vs +0.88 (±1.84) kg (P =.01)  Diet  Improvement in the intervention group (P<0.01)  Diabetes medication: NS  Physical activity: improvement in the intervention group but no difference between groups | |

| Study,  Country | Population targeted | Longest Follow up (months) | Experimental intervention  Strategy  description | Control intervention | Outcomes | Main results  (experimental versus control) |
| --- | --- | --- | --- | --- | --- | --- |
| DePue 2013[18] American Samoa, USA  Cluster RCT |  | 12 | self-management education (n=104) delivered by trained NCM and CHW (training on guidelines, chronic care model, patient-centered communication skills) for Hispanic Americans. | usual care (n=161): waiting list + education material | HbA1c, BP, BMI, waist circumference, | HbA1c  Significan reduction among CHW participants, compared with usual care  Adjusted MD^=-0.53% (p<0.03)  BP, BMI, waist circumference  No significant differences between groups.  Trend for individuals with more contacts |
| Garcia 2015 [57]  Texas, USA | Mexican Americans  Mean age: 49.6 years  Women:67%  45%  Spanish-speaking | 6 | Culturally tailored self management education (n=39): 8 weekly in-home, interactive,  educational sessions  delivered by bilingual registered nurse | Usual health care (n=33): counseling + education from regular health care providers | HbA1C, cholesterol, LDL, BP, BMI, diabetes knowledge, quality of life | Total Cholesterol  Significant improvement for the intervention group  (p=0.003)  LDL  Significant improvement for the intervention group  (p=0.014)  Self-efficacy (p=0.008)  Significant improvements in HbA1c, DBP, HDL and quality of life in both groups but no difference between groups.  Improvements in number of symptoms, symptom severity, diabetes knowledge, empowerment,  although were not sustained at 6 months  SBP, triglycerides and BMI: NS |

| Study,  Country | Population targeted | Longest Follow up (months) | Experimental intervention  Strategy  description | Control intervention | Outcomes | Main results  (experimental versus control) |
| --- | --- | --- | --- | --- | --- | --- |
| Gary 2009 [51]  Baltimore,  USA | African Americans  Intervention: 54% mean age: 58 years  women:73% | 24 | Intensive (Project Sugar2 n=269): minimal intervention+individualized culturally-tailored care provided by a nurse case manager and a community health worker + clinical algorithms with feedback to primary care providers  Low and high frequency | Minimal intervention (n=253): telephone-based intervention by a lay health worker + remind (by telephone calls every 6 months) + education material by email | HbA1C, cholesterol, BP, BMI, ED visits, hospital admissions | HbA1C (high frequency intervention), HDL, DBP  Improvements in experimental group from baseline  Other clinical outcomes: n.s.  ED visits (overall intensity)  RR 0.77 (95% CI 0.59 to 1.0)  ED visits High frequency intervention)  RR= 0.66; 95% Cl, 0.43-1.00;  Hospitalizations: NS  Retention rate  High follow up rate |
| Kim 2009 [52]  Washington, USA | Korean American immigrants  Mean age:53.8 years  Women:44.4% | 7.5 | Community based  self-help intervention program  (n=40): education sessions at a community site delivered by a bilingual nurse and home glucose monitoring with tele-transmission. Telephone counseling | delayed intervention (n=39) | HbA1c, cholesterol, BP, BMI, diet, physical activity, glucose monitoring, diabetes knowledge | HbA1c change  -1.3% vs -0.4% (p=0.01)  Cholesterol  -24.7 mg/dl vs 7.2 mg/dl  (p=0.03)  Triglycerides  -84.6 mg/dL vs -4.2 mg/dL (p<0.05)  No differences between groups for other clinical outcomes  Intervention group showed greater improvements in diabetes knowledge, self-care activities, self-efficacy, attitudes towards diabetes and quality of life |

| Study,  Country | | Population targeted | | Longest Follow up (months) | | Experimental intervention  Strategy  description | | Control intervention | | Outcomes | | Main results  (experimental versus control) | |
| --- | --- | --- | --- | --- | --- | --- | --- | --- | --- | --- | --- | --- | --- |
| Kim 2015 [46]  Texas-Mexico, USA | | Korean American immigrants  Mean age:58,7 years  Women:43% | | 12 | | Self-management (n=129): group sessions education delivered by CHW + follow-up phone calls  Program based on Predisposing, Reinforcing, and  Enabling Constructs in Education/environmental Diagnosis and  Evaluation (PRECEDE)–Policy, Regulatory, and Organizational  Constructs in Educational and Environmental Development  (PROCEED) model | | Educational material (n=130) based on self-management principles and information about resources in the community | | HA1C, glucose levels, cholesterol, LDL, BP, triglycerides, diabetes knowledge, quality of life | | HbA1c% change  Improvements in both group, reductions in the intervention group greater than control group  -1.3% vs -0.7% (p<0.001)  MD=-0.6% (p<0.001)  Glucose levels  MD= –24.1 (p<0.01)  Total cholesterol, LDL  Improvements in both group, but no difference between group  No difference between groups for BP and triglycerides  Diabetes knowledge changes from baseline  MD=2.1 (p<0.001)  DM quality of life change  Mean=8.6 (p<0.001)  Attitudes toward diabetes  3.6 (p<0.001) | |
| Hotu 2010 [22]  Auckland,  New Zealand | | Maori and Pacific New Zealanders  Mean age: 61.5 years  Women: 46% | | 12 | | Community care (n=33): Home visits by a nurse health care assistant and additional services (transport to pharmacy, laboratory and clinical appointments) | | usual care (n=32) from family doctor + education materials | | BP, A1C, 24-h urine protein excretion, cholesterol, cardiac parameters of LV mass/BSA, LA volume/BSA and E/E’.  death | | SBP  Significant improvement  149 mmHg vs 140 mmHg (P <0.05)  No significant differences were seen between the groups in cardiovascular outcomes.  number of  antihypertensive medications  3.4 ± 1.1 vs 2.3 ±1.0 (P < 0.05)  urine protein excretion (mean)  1.95 vs 2.20 (p<0.005)  No differences were seen between the  groups DBP, serum creatinine level,  HbA1c or total cholesterol  Retention rate  87.5% vs 91% | |

| Study,  Country | | Population targeted | | Longest Follow up (months) | | Experimental intervention  Strategy  description | | Control intervention | | Outcomes | | Main results  (experimental versus control) | |
| --- | --- | --- | --- | --- | --- | --- | --- | --- | --- | --- | --- | --- | --- |
| Liou 2013 [53]  Taiwan | | Underserved, rural setting  Mean age: 56.8 years  Women:50% | | 6 | | Shared care team (n=54): 4 sessions of diabetes education + 2 interactive online sessions delivered by a nurse, dietician and diabetes specialist | | usual care (n=41): individual education session delivered by a licensed practical nurse | | HbA1C, BMI, BP, cholesterol | | HbA1c mean change  0.7± 1.3% vs 0.1 ± 1.0% p<0.03 after adjustment for baseline HbA1c  No significant differences  in lipid profiles and blood pressure, BMI changes between groups | |
| Lujan 2007 [54]  Texas, USA | | Mexican Americans  Mean age: 58 years  Women:80% | | 6 | | Self-management (n=75): 8 week group education classes and telephone follow up delivered by trained promotoras (bilinguas clinic employee) | | Usual care (n=74): patient education delivered by clinic staff during follow up visits | | HbA1C, diabetes knowledge | | HbA1C  Greater Improvements in the intervention group than in control group (  -0.45% vs 30%;P < .002)  diabetes knowledge  Improvements of DKQ score higher than the control group (P < .002)  Retention rate  96% | |
| Lynch 2014[59] Chicago,  USA | | African Americans  Mean age:54.1 years  Women: 67.1% | | 6 | | Culturally self management program (LIFE program) (n=30): group classes led by a register dietitian + weekly telephone calls from trained peer-supporters | | Self management classes (n=31) 3-hours group classes led by a community health worker | | Proportion of pz who achieved a 5% weight loss and 0.5% reduction of HbA1c  HbA1C levels, weight, BP, BMI, diet, physical activity, medication adherence | | weight ≥5%:  NS  HbA1c ≥0.5% reduction:  50% v. 21.4% (p=.03)  Weight change, HbA1c, BP  Improvements for the intervention group but no difference between groups.  Significant improvements in general diet (n° days in past week)  MD= 1.9 (p<0.001)  and specific diet  MD=1.2 (p<0.02)  changes in calories from protein  MD=2.4 (p<0.01)  Physical activity  MD=2.517 Kcal (p<0.01)  Retention rate: 83.7% vs 93.5% | |

| Study,  Country | | Population targeted | | Longest Follow up (months) | | Experimental intervention  Strategy  description | | Control intervention | | Outcomes | | Main results  (experimental versus control) | |
| --- | --- | --- | --- | --- | --- | --- | --- | --- | --- | --- | --- | --- | --- |
| McDermott 2015*[19]  North Queensland,  USA  Cluster RCT | | Australian indigenous, low-income  Mean age: 4.9 years  Women: 62%  Unemployed: 46.5% | | 18 | | Case management (n=100) from a community-based health worker supported by a clinical outreach team+home visits | | wait-list control with usual care (n=133) | | HbA1C, BP, BMI, cholesterol, GP Management  Plan (GPMP) for diabetes, appropriate medication use, glucose monitoring, eye check, quality of life | | HbA1c (mean change)  Significant improvement  −1.0% vs −0.2% (p = 0.02)  Intervention group participants were more likely to receive nutrition and dental services according to scheduled care plans.  Small improvements in both groups for total cholesterol, LDL, HDL ratio, with slightly better results in the intervention group.  Retention rate: 83% vs 96% | |
| Prezio 2014[49] Texas, USA | | Mexican Americans  Mean age:54 years  Women:67.2%  Hispanic:81.6% | | 12 | | Culturally tailored diabetes education program (n=90) led by a trained CHW+ medical care | | usual care (n=90): medical care+ education material | | HbA1C, BP, BMI, cholesterol | | HbA1c  MD=-0.7% (95%CI -1.2, -0.1)  Other outcomes:  No differences between groups  Retention rate: 84% vs 84% | |
| Palmas 2014[31]  New York,  USA | | Hispanics  Mean age=57.6  Women=62%  Hispanic: 100% | | 12 | | Tailored intervention (n=181): case management+visits + group visits (10 sessions) focused on nutrition education+ telephone follow-up delivered by community health workers | | usual care (n=179) from primary care provider + educational materials + phone calls | | HbA1C, BP, LDL | | HbA1c  Modest reduction in the intervention group but no significant differences between groups (p=0.131).  BP and LDL  NS  Phone calls vs in person contacts shows a significant reduction in HbA1C levels (P = 0.04)  people with glycemic control (baseline A1C <7%) shows a significant reduction in HbA1C levels  Retention rate: 81.4% vs 88% | |

| Study,  Country | | Population targeted | | Longest Follow up (months) | | Experimental intervention  Strategy  description | | Control intervention | | Outcomes | | Main results  (experimental versus control) | |
| --- | --- | --- | --- | --- | --- | --- | --- | --- | --- | --- | --- | --- | --- |
| Partapsingh 2011 [23]  South Trinidad | | low socio-economic status  Women:35%  50-59 years: 44%  20% occasionally employed  Total monthly income <1000$ | | 12 | | Stages of Change model. Case management (n=61): Individualized and personalized care based on patient’s stage | | usual care (n=61) | | A1C, BMI, BP, cholesterol, triglycerides, diet, physical activity, medication adherence | | HbA1c  MD =-0.57%  (95%CI 0.07 - 1.07).  No significant difference between groups for other outcomes  Retention rate: 95% vs 100% | |
| Perez-Escamilla 2015[47]  USA | | Latino  Mean age: 56 years  Women: 73.5% | | 18 | | Culturally tailored counseling (n=195): 17 home-based education sessions (DIALBEST program) delivered by CHW | | standard care (n=106): glucometer and prescription for glucose test trips + education | | A1C, BMI, fasting glucose, cholesterol, diet, physical activity, medication adherence, glucose monitoring | | HbA1c  MD= -0.55% (-0.96 to -0.14)  change%  MD= -5.52%  (95% CI -8.93, -2.11% )  Fasting glucose:  MD=-1.08 mmol/L  [95% CI 21.79, 20.39 mmol/L], P =0.002)  No significant difference between groups for other outcomes  Retention rate  75% vs 65% | |
| Rothschild 2014 [30]  Chicago,  USA | | Mexican Americans  Mean age:53,7 years  Women:67. 4% | | 24 | | Self-management training at home (n=73) (MATCH program): delivered by a trained, bilingual CHW | | Educational material (bilingual) (n=71) | | HbA1C, % achieving BP control, medication adherence, physical activity, diet, glucose monitoring | | HbA1C  MD=- 0.69% (95%CI-0.95%, -0.15)  BP, medication adherence, glucose monitoring: NS  Physical activity:  Improvement in the intervention group  Improvement in self-efficacy and in fruit and vegetable consumption but no significant difference between  arms  Retention rate: 79.5% vs 85.9% | |

| Study,  Country | | Population targeted | | Longest Follow up (months) | Experimental intervention  Strategy  description | Control intervention | Outcomes | Main results  (experimental versus control) |
| --- | --- | --- | --- | --- | --- | --- | --- | --- |
| Ruggiero 2010[72] Chicago,  USA | | Minority (African American, Latino), low-income  Mean Age:65,8  Women:66% | 6 | Patient education (face-to-face clinical contacts, telephone contacts, and clinic visits) (n=25) delivered by medical assistant with specific training in diabetes care and behavioral coaching | usual care with 2 brief (<30 minutes) sessions + follow up telephone calls (n=25) | HbA1c, psychosocial measures | *HbA1c*  Improvement in intervention group but no difference between groups  Increase in perceived empowerment  and reduction in perceived diabetes related problems  Retention rate  Not reported | |
| Shea 2009 [45]  New York,  USA | | Ethnically diverse, medically underserved  Mean age: 71 years  Women: 63% | | 60 | home telemedicine unit for self/monitoring and communication with nurse case manager (n=844) | usual care (n=821), received clinical care from their primary care providers, without other guidance or direction from study personnel. | HbA1C, LDL, BP, all mortality | HbA1C  MD= -0.29% (95%CI -0.46, -0.12)  LDL (at 4 years)  MD= -5.83 mg/dL (95%CI -9.16, -2.50]  SBP  MD=-4.32 mmHg [95%CI -6.72, -1.92]  DBP  MD=-2.63 mmHg[95%CI -3.74, -1.52]  Deaths: 176 vs 169  No significance difference between groups  Retention rate: 40% vs 56.4% |
| Sixta 2008 [28]  Texas,  USA | | Mexican-Americans  Mean age:54 years  Women:71% | | 6 | self-management education (n=63): 10-week education group sessions and follow-up by promotores in consultation with a care team | Usual care (n=68) delivered by provider at the clinic or to a self-care  management | HbA1C, knowledge, beliefs | HbA1C  No difference between groups  Intervention  group decreased 0.04 and no change in control group  At 6 months, the HbA1c levels in both groups increased  (0.18 and 0.05, respectively).  DKQ, HBQ  No difference between groups  Retention rate: 80% |

| Study,  Country | | Population targeted | Longest Follow up (months) | Experimental intervention  Strategy  description | Control intervention | Outcomes | Main results  (experimental versus control) |
| --- | --- | --- | --- | --- | --- | --- | --- |
| Spencer 2011 [48]  Detroit,  USA | African Americans, Latinos  Mean age:52.5 years  Women: 43%  57% African Americans | 6 | Culturally tailored self-management education (n=94): group classes delivered from CHW + home visits + clinic visit | delayed-intervention (n=99) | A1C, LDL, BP, physical activity, diet, glucose monitoring, medication adherence | HbA1c  %change between intervention and control groups = –9.7(–15.9, –3.0,  (p < 0.01)  LDL  Improvement in the intervention group. No differences between group  Other outcomes: NS  Retention rate: 84% vs 85% | |
| Tang 2014[40] Detroit,  USA | Latino, low-income mean age: 49.3 years  Women: 58.6%  Spanish speaking: 100% | 18 | Diabetes self management education (n=60): 6-month of education + weekly group sessions delivered by Peer leader with telephone outreach to those unable | self management program (n=56): 6-month DSME program + monthly telephone outreach delivered by Community health workers | A1C, BP, BMI, knowledge in social support | HbA1c  Improvement in the intervention group.  No differences between  groups at any time point  Other outcomes: n.s.  Changes in perceived  diabetes social support over time for both groups. Difference between group at 6 and 12 months  Retention rate:  62% vs 57% | |
| Thom 2013 [50]  San Francisco,  USA | low-income  mean age: 55 years  Women:61%  Income (per year)  <$10,000:53% | 6 | Self-management (n= 148) delivered by trained peer health coaches outside the clinic by telephone or during visits | usual care n(151): all services normally available including access to a nutritionist and diabetes educator | HbA1C, LDL, SBP, BMI  % patients with decrease in HbA1c ≥1.0% and HbA1c <7.5% | HbA1C  MD=- 0.69% (p<0.01)  Decrease in HbA1c ≥1.0%  49.6% vs 31.5% (p<0.001)  HbA1c <7.5%  22% vs 14.9 (p<0.04)  No significant difference  in change for LDL-C, SBP and BMI  Retention rate: 94,6% vs 89,4% | |

| Study,  Country | | Population targeted | | Longest Follow up (months) | | Experimental intervention  Strategy  description | | Control intervention | | Outcomes | | Main results  (experimental versus control) | |
| --- | --- | --- | --- | --- | --- | --- | --- | --- | --- | --- | --- | --- | --- |
| Tobe 2006[60]  Saskatchewan,  Canada | | Native population, First Nations people  Mean age:55.5 years  Women:61.5% | | 12 | | community-based treatment (n=50): home care nurses using a predefined treatment algorithm | | usual care (n=49): home case nurse | | BP, A1C, BMI, cholesterol, adverse events (complications, hospitalizations) | | No differences between groups  Retention rate: 96% vs 96% | |
| Willard-Grace 2015[73]  San Francisco,  USA | | Low-income, Latino  Mean age:52.7%  Women: 55.3%  Latino: 70%  <$5000:34% | | 12 | | Self-management education (n=224): delivered clinic-based medical assistant health coaching | | usual care (n=165): access to any resources available at the clinic | | Proportion of patients reaching a composite measure,  HbA1C, BP, cholesterol | | % composite measures  46.4% vs 34.3%, P = .02)  Secondary composite measure of reaching all clinical goals (34.0% vs 24.7%, p =0.05).  Differences in the primary and secondary composite goals and in 2 of 3 secondary goals (improvement in HbA1c and LDL cholesterol) were significant at clinic site A but not at clinic site B.  SBP: NS  Retention rate: 93% vs 84% | |

*adjusted for clustering and confounding;

^adjusted for church, comorbidities, perceived diabetes control, benefits of control, Patient Activation Measure, history of doctor visits, gender, age, baseline

risk level.

NS=no significant difference between groups; NR=not reported; OGLA= prescription of at least one oral glucoselowering agent; MPR= medication possession ratio; BMI=Body Mass Index; LDL=low density cholesterol; BP=blood pressure; CDSMP =Chronic Disease Self-Management Program; PDA=personal digital assistant; SDCA=Summary of Diabetes Self-care Activities Questionnaire; AADQ=Acceptance and Action Diabetes Questionnaire value presented in mean; NCM=Nurse case manager; CHW=community health workers; DDP=Diabetes Prevention Program;
